# Gaining Insight into Mitochondrial Targeting: AUTAC-Biguanide as an Anticancer Agent

**DOI:** 10.3390/molecules29163773

**Published:** 2024-08-09

**Authors:** Julie Vatté, Véronique Bourdeau, Gerardo Ferbeyre, Andreea R. Schmitzer

**Affiliations:** 1 Département de Chimie, Faculté des Arts et des Sciences, Université de Montréal, 1375 Av. Thérèse Lavoie-Roux, Montréal, QC H2V 0B3, Canada; 2 Département de Biochimie et Médecine Moléculaire, Université de Montréal, Montréal, QC H3C 3J7, Canada; 3 Montréal Cancer Institute, Centre de Recherche-Centre Hospitalier de l’Université de Montréal (CR-CHUM), Université de Montréal, Montréal, QC H2X 0C1, Canada

**Keywords:** AUATAC, biguanide, mitochondria, autophagy

## Abstract

AUTAC-Biguanide is a hybrid compound designed to target mitochondria, inducing their degradation by mitophagy. This study unveils the potential of biguanides as cancer cell-targeting agents, emphasizing AUTAC-Biguanide’s superior antiproliferative properties compared to metformin and its selectivity for cancer cells. The mechanism behind this heightened effect includes the ability of AUTAC-Biguanide to trigger mitophagy. By providing a comprehensive analysis of these findings, this study adds valuable insights to the field of mitochondrial-targeting anticancer agents.

## 1. Introduction

The influence of biguanide derivatives on the mitochondrial respiratory chain has become a well-established area of research. Their antitumor properties, to some extent, stem from their ability to inhibit oxidative phosphorylation [1]. While numerous studies have demonstrated the inhibition of complex I in response to metformin and phenformin treatments [2], the precise molecular target of biguanides within the mitochondria remains unclear [3,4]. Furthermore, some studies indicate a lack of biguanide activity on isolated mitochondria, suggesting that these compounds may only exert their effects within intact cells [5]. One plausible explanation is that these compounds do not directly interact with mitochondria [4]. Another hypothesis suggests that the action of biguanides is, indeed, the result of a direct interaction, but is triggered by their accumulation within these organelles [4,5,6]. This raises an intriguing question regarding whether biguanides primarily target mitochondria themselves or if their target is actually in the cytoplasm, the inhibition of oxidative phosphorylation being an indirect consequence of this interaction.

The propensity of monosubstituted biguanides to localize within mitochondria primarily hinges on their protonation status at physiological pH. Positively charged biguanides exhibit an affinity for the negatively charged mitochondrial inner membrane [7,8]. Since the inception of research on biguanide derivatives in the 1920s–1930s, limited attention has been given to exploring their interactions within mitochondria [9]. A notable breakthrough occurred in 2018, when a study demonstrated the co-localization of these compounds with cytochrome c [10]. In pursuit of this understanding, the researchers conducted an in vitro experiment where they conjugated a metformin derivative with a fluorescent marker using a copper-catalyzed azide–alkyne cycloaddition method applicable within intracellular environments. Their observations revealed the accumulation of the metformin derivative in mitochondria.

Nevertheless, several crucial questions persist. Does the observed accumulation in mitochondria suffice to designate mitochondria as the primary target of biguanides? Furthermore, does incorporating a biguanide function into a compound redirect its mechanisms of action toward mitochondria? To delve into the interaction between biguanidium salts and mitochondria, and with the aspiration of formulating a potentially more potent anticancer agent than metformin, we fused metformin with a ligand capable of activating the cell’s autophagy system. The effects of this hybrid compound on mitochondrial structure may offer compelling evidence supporting the notion that biguanides indeed target mitochondria.

**Figure 1:**
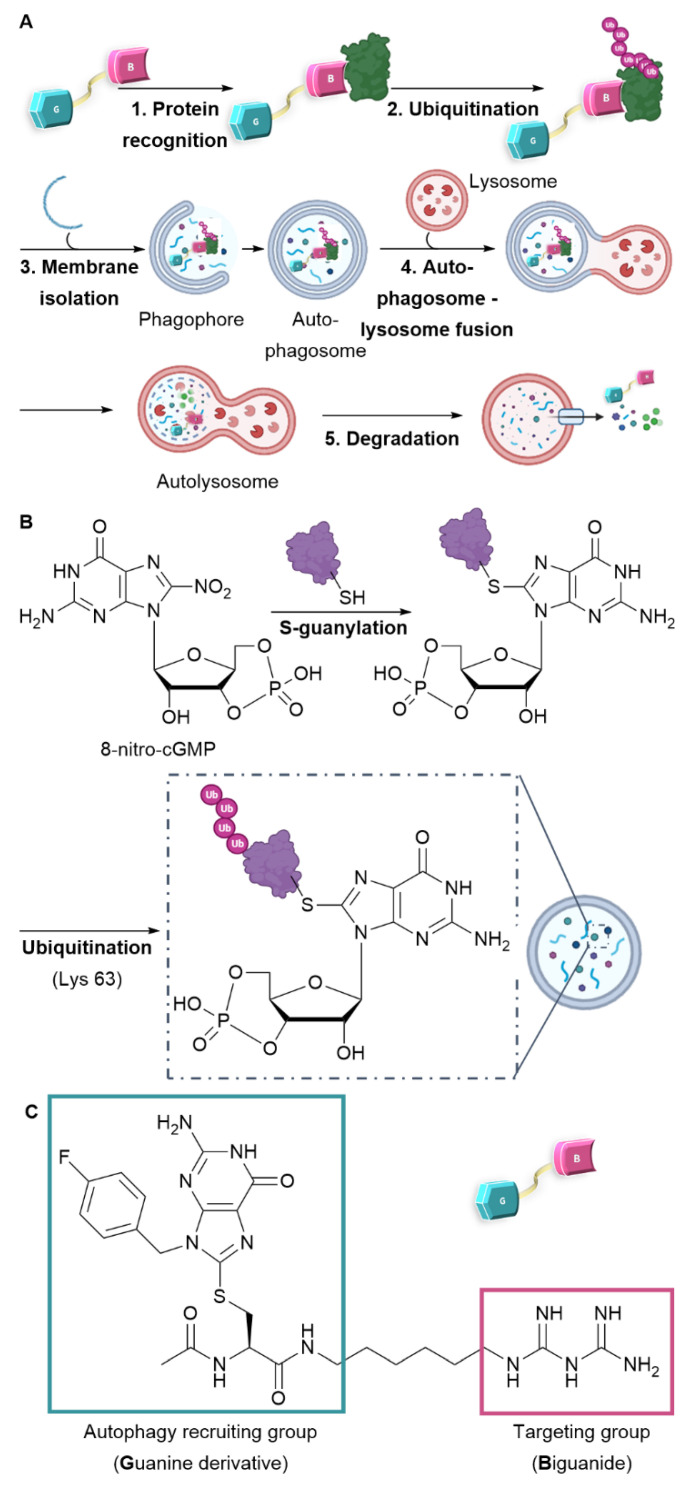
Design and mechanism of AUTAC-Biguanide. (**A**) Mode of action of the AUTAC compound and the mechanism of autophagy. (**B**) S-guanylation and ubiquitination protein recognition mode for selective degradation by autophagy. (**C**) Design and structure of AUTAC-Biguanide.

An emerging strategy involves promoting the degradation of a target molecule by attaching it to a ligand that can harness the cell’s intrinsic degradation pathways [11,12,13]. Autophagy is a cellular mechanism that facilitates the degradation of a broad spectrum of substrates, ranging from individual proteins to entire organelles. This process operates through the selective tagging of proteins and their subsequent incorporation into autophagosomes. Upon fusion with lysosomes, these autophagosomes allow the enzymatic degradation of their contents by hydrolases [14]. To specifically target proteins or organelles of interest using this mechanism, recent developments have led to the creation of bifunctional molecules, including AUTACs (Autophagy Targeting Chimeras) and ATTECs (Autophagosome Tethering Compounds) [11,12,15]. These molecules consist of two essential components: one ligand targeting the protein to be degraded, represented by the pink motif (Biguanide in our case), and another ligand that recruits the degradation system, denoted by the blue motif (Guanine-derivative) (see Figure 1A). Notably, the creation of an ATTEC compound with mitochondria-targeting capabilities has already been successfully realized through the incorporation of a triphenylphosphonium (TPP) targeting group [16]. Liu et al. have successfully showcased the capacity of ATTEC–TPP derivatives to trigger mitophagy [17]. While TPP is commonly employed to direct molecules toward mitochondria, it has the drawback of being highly apolar, making conjugation with excessively hydrophobic molecules a challenge due to resulting insolubility in biological media. The biguanide function, being highly polar, offers an alternative to TPP. Conjugation with hydrophobic molecules strikes a balance between hydrophilicity and hydrophobicity, ensuring both the diffusion of molecules across cell membranes and their solubility.

Among the diverse approaches to activate autophagy, the creation of AUTAC compounds draws inspiration from protein S-guanylation as a triggering mechanism. In fact, a study conducted by Arimoto et al. in 2013 emphasized the significance of S-guanylation as a marker that induces bacterial autophagy within the cytoplasm of mammalian cells [18,19]. This process is characterized by the interaction between the compound 8-nitro-cGMP (Cyclic Guanosine Monophosphate) and a protein’s cysteine residue. The creation of a thioether bond facilitates the subsequent attachment of ubiquitin through lysine 63 (K63), thereby marking the protein for degradation by the lysosome [20]. As a result, the ubiquitin chain is subsequently identified by autophagy receptors and integrated into the autophagosome (as illustrated in Figure 1B). Taking inspiration from this recognition mechanism, Arimoto et al. achieved the synthesis of the initial AUTAC compound. This one comprises a guanine derivative and a mitochondria-targeting ligand, and has demonstrated the ability to induce mitophagy [15].

The research presented below intends to confirm the mitochondria-targeting potential of biguanides and enhance their efficacy as anticancer agents. We designed an AUTAC-Biguanide compound by incorporating a biguanide functional group as a targeting moiety to the guanine motif, known for its ability to recruit autophagy, as originally reported by Arimoto et al. [15]. The two ligands connected by a carbon chain (Figure 1C) were assessed for their capacity to target and modify mitochondria and inhibit the proliferation of cancer cells in vitro.

## 2. Results and Discussion

**Scheme 1:**
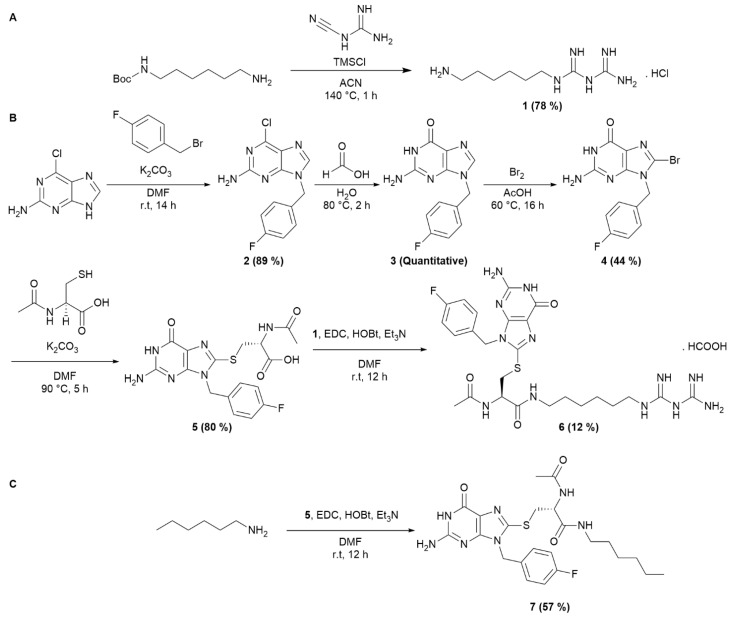
Synthesis of AUTAC-Biguanide and its control, AUTAC-Hexyl. (**A**) Synthesis of intermediate **1**. (**B**) Synthesis of AUTAC-Biguanide **6**. (**C**) Synthesis of AUTAC-Hexyl **7**.

The synthesis of the AUTAC-Biguanide was the result of the conjugation of two building blocks. *N*-Boc-1,6-hexanediamine was introduced in stoichiometric quantities with dicyandiamide and TMSCl to form compound **1** in salt form, by in situ deprotection of the *tert*-Butoxycarbonyl (Boc) group (Scheme 1A). To form the guanine ligand, the synthetic route previously described by Arimoto et al. [15] was revisited with the aim of reducing the number of reaction steps. By substituting 4-fluorobenzyl alcohol with fluorobenzyl bromide, it was possible to streamline the synthesis process by eliminating three steps, reducing it to just four steps. The initial step involved the formation of compound **2** through an SN2 reaction involving the purine imidazole and fluorobenzyl bromide, yielding the desired isomer with an 89% yield. The subsequent addition of formic acid led to the complete formation of intermediate **3**, followed by bromination to yield compound **4**. This one then reacted with acetylcysteine thiol to yield guanine derivative ligand **5**, incorporating an essential carboxylic acid function to enable coupling with the previously synthesized biguanide derivative **1**. After HPLC-prep purification, compound **6** was successfully obtained with a 12% yield, as depicted in Scheme 1B. A control derivative was also synthesized without the biguanide function. In this case, compound **5** was coupled with hexylamine to retain only the guanine ligand and the spacer, following the synthetic route depicted in Scheme 1C. This approach offers the advantage of simplicity, requiring only a few steps.

To evaluate its potential as an anticancer agent, AUTAC-Biguanide **6** underwent testing on two pancreatic cancer cell lines, namely KP4 and PANC1. Its effectiveness was subsequently compared with that of metformin, as well as guanine derivatives AUTAC-COOH **5** and AUTAC-Hexyl **7**. The results showed that controls **5** and **7** had no discernible impact on the growth of cancer cells, while metformin exhibited toxic activity at concentrations ranging from 1 to 4 mM. Notably, the combination of the AUTAC ligand with the biguanide proved significantly more potent, demonstrating a median effective concentration of approximately 0.1 mM. This concentration is ten times lower than that of metformin, highlighting the compound’s potential antitumor properties (see Table 1). The compounds were also tested on two healthy cell lines, hTERT-HPNE (immortalized pancreatic cells) and IMR90 (lung fibroblasts), to assess their toxicity. Metformin had no noticeable effect on either cell line at the concentrations examined. Conversely, the controls, AUTAC-COOH **5** and AUTAC-Hexyl **7**, appeared to affect hTERT-HPNE without exhibiting toxicity toward IMR90. AUTAC-Biguanide, much like metformin, had no adverse impact on any of the healthy cell lines. However, it is important to note that determining the EC_50_ (effective concentration reducing cell viability to 50%) for these compounds became challenging as the solubility of the compound exceeded 1 mM in the culture medium (see Table 1). Both AUTAC-Biguanide **6** and metformin exhibited a remarkable selectivity for cancer cells over healthy cells, indicating the potential of the biguanide function as a cancer cell-targeting mechanism. These derivatives demonstrated a selectivity index greater than 10, establishing them as promising anticancer agents.

**Figure 2:**
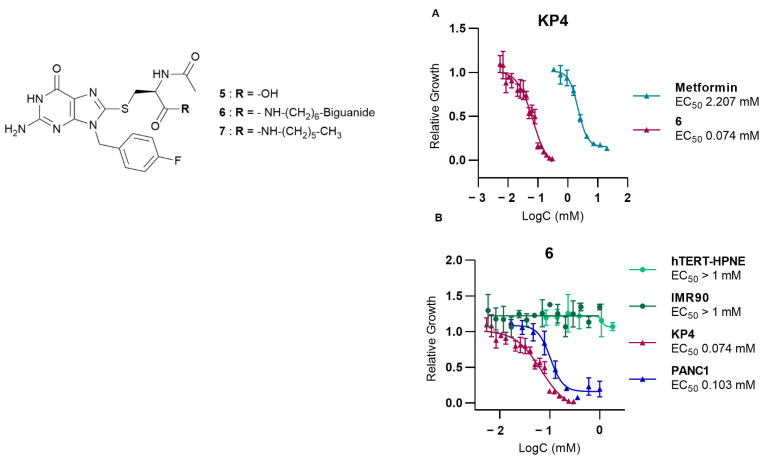
Study of the effect of AUTAC-Biguanide **6** on different cell lines. (**A**) Effect of **6** on pancreatic cancer cells KP4 compared to metformin (**B**) Effect of **6** on the proliferation and growth of KP4 and PANC1 (cancer cells), and hTERT-HPNE and IMR90 (healthy cells), to assess its selectivity for cancer cells versus healthy cells.

Notably, compound **6** outperformed metformin, as its anticancer effects were observed at lower concentrations, as illustrated in Figure 2. Evidently, AUTAC-Biguanide stands out from metformin due to its significantly improved antiproliferative properties in cancer cells. To gain insights into the underlying reasons for this enhanced effect, a comprehensive investigation of the compound’s mechanism of action was undertaken and subsequently compared with its biguanide and guanine controls.

**Figure 3:**
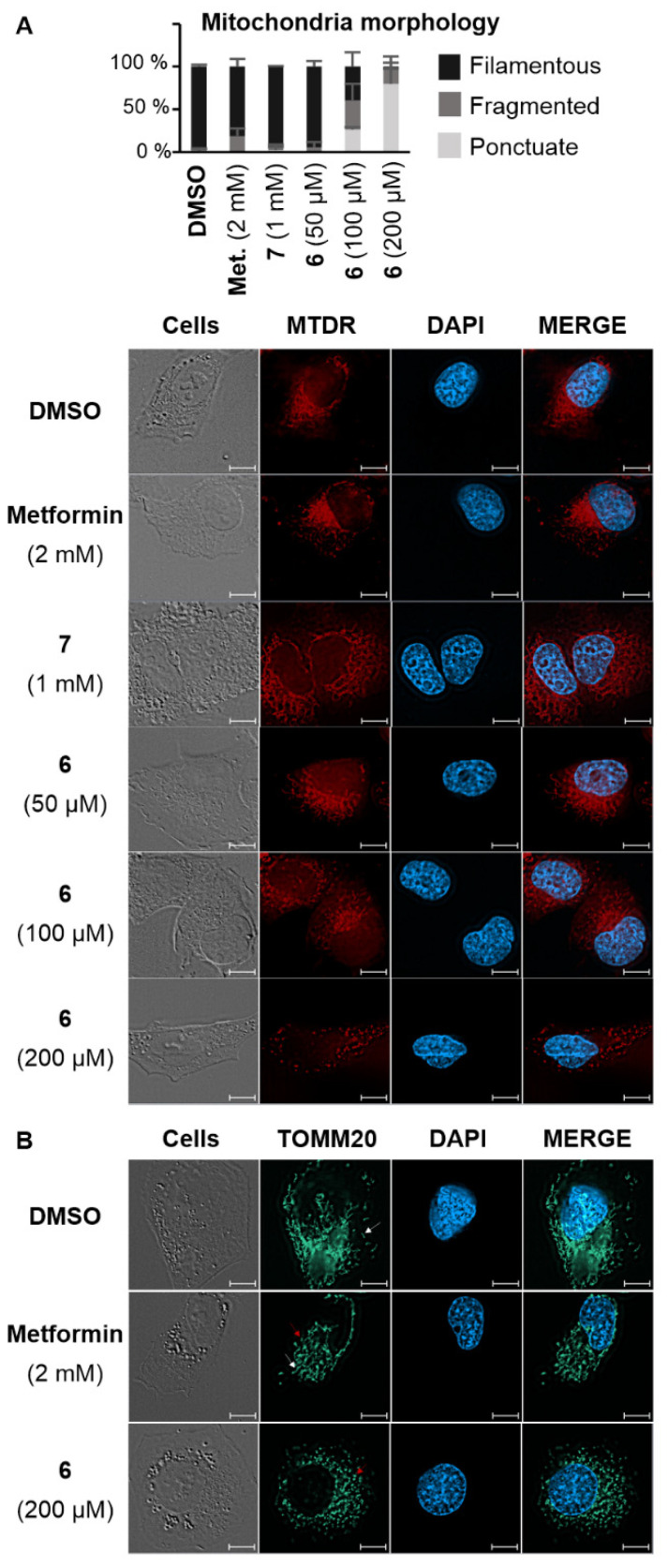
Confocal microscopy images of KP4 pancreatic cancer cells following treatment with AUTAC-Biguanide **6** or controls. (**A**) Morphology of KP4 cells after 24 h of treatment with DMSO, metformin (2 mM), compounds **7** (1 mM), or **6** (50 μM, 100 μM, and 200 μM), and staining of mitochondria with the Mitotracker^®^ Deep Red FM (MTDR) fluorescent probe and the nuclei with the fluorescent dye DAPI; cell morphology counts performed on a minimum of 100 cells per treatment (n > 2). (**B**) Observation of KP4 cells after 24 h of treatment with DMSO, metformin (2 mM), or compound **6** (200 μM) by immunofluorescence of the TOMM20 protein performed with the combination of antibodies against TOMM20 and an anti-mouse Alexa Fluor 488. The nuclei were stained with the fluorescent dye DAPI. Scale bars: 10 µm.

As the impact of biguanide compounds is primarily linked to mitochondria, a study was conducted to investigate whether the guanine motif had the potential to induce autophagy in these organelles. In healthy cells, mitochondria form a dynamic network that evolves throughout the cell cycle. This network presents itself as a collection of mobile filaments, which tend to undergo fragmentation at the onset of apoptosis, resulting in a disorganized network with a punctate appearance [21,22]. The morphology of mitochondria can be influenced by various stimuli, which may have a more or less direct impact on these organelles. These alterations can be visualized using confocal microscopy. Depending on their morphology, mitochondria can be categorized as filamentous, fragmented (representing an intermediate state with both filaments and dots), or punctate [21,22]. A fluorochrome that specifically targets mitochondria (MitoTracker Deep Red FM or MTDR) was employed to examine the mitochondrial morphology in KP4 cells [23]. In the presence of metformin and AUTAC-Hexyl **7**, mitochondria predominantly formed a filamentous network. However, upon treatment with AUTAC-Biguanide, they exhibited a punctate configuration. Notably, the extent of this effect was concentration-dependent and became evident only when concentrations exceeded the EC_50_ threshold (100 µM). At this concentration, more than 50% of cells displayed a fragmented or punctate mitochondrial network within just 24 h of treatment. Doubling this concentration resulted in fewer than 10% of cells maintaining a filamentous network. Moreover, there was a decrease in fluorescence intensity, indicating potential mitochondrial degradation, which aligns with previous findings (Figure 3A). To enhance image resolution and validate the disparity in the effects of metformin and AUTAC-Biguanide, immunostaining was performed. In this context, an immunofluorescent label was applied to TOMM20, a mitochondrial outer membrane protein. The results were consistent with MTDR results, showing that the majority of cells assumed a punctate appearance after treatment with 200 µM of compound **6**, while 2 mM of metformin induced a mild fragmentation of the network, characterized by the presence of filaments (indicated by white arrows) and dots (indicated by red arrows) (Figure 3B). Furthermore, mitochondria seemed to adopt the same morphology with both AUTAC-Biguanide and ATTEC–TPP treatments as reported by Liu et al. [17], suggesting that both compounds have the same mode of action of inducing mitophagy.

**Figure 4:**
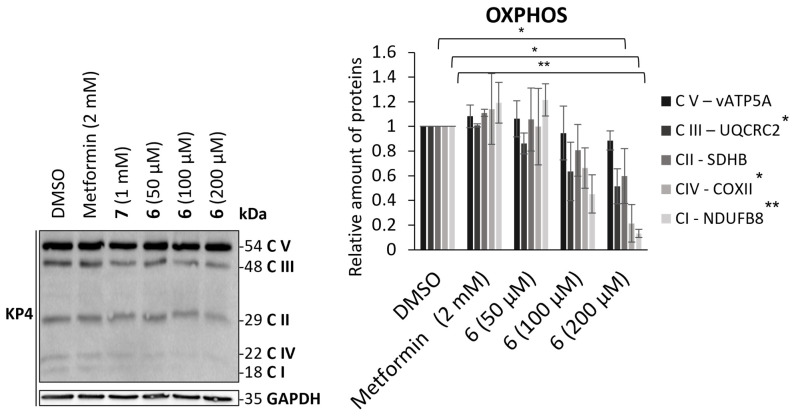
Effect of AUTAC-Biguanide **6** and its controls on OXPHOS proteins levels in KP4 after 24 h of treatment and relative quantification of protein levels for metformin and AUTAC-Biguanide (**6**); * *p* ≤ 0.05; ** *p* ≤ 0.01 (ANOVA).

To further assess mitochondrial mass reduction induced by the action of compound **6**, its effect on the levels of proteins belonging to the mitochondrial respiratory chain was studied (Figure 4). To this end, protein transfer was performed following 24 h of treatment of compound **6** and metformin in KP4 cells. A cocktail of antibodies was used to reveal and quantify various mitochondrial proteins involved in oxidative phosphorylation (OXPHOS). This mixture enabled the detection of one protein from each of the respiratory chain complexes: vATP5A (complex V), UQCR2 (complex III), SDHB (complex II), COXII (complex IV), and NDUFB8 (complex I). While metformin did not appear to affect the levels of the proteins studied, a decrease was visible upon treatment with compound **6** for the majority of proteins, with the exception of vATP5A and SDHB. The mechanism of action of compound **6** is different than metformin’s, since compound **6** induces a decrease in complex I and complex IV mitochondrial respiratory chain proteins, which is not the case for either metformin or compound control **7**. The addition of a guanine function therefore enhances the efficacy of metformin as an anticancer agent by modifying its mechanism of action. However, it is not yet possible to state with certainty that guanine induces autophagic degradation of the biguanide target, and since guanine **7** alone has no effect on OXPHOS proteins, AUTAC-Biguanide **6** directs its action toward mitochondria.

To delve deeper into the mechanism of action of AUTAC-Biguanide **6**, investigations were conducted to ascertain whether autophagy played a role in inducing mitochondrial degradation. The autophagic process hinges on the formation of autophagosomes, which encapsulate proteins or organelles. These autophagosomes subsequently fuse with lysosomes, leading to the degradation of their contents [24]. One of the most widely used markers of autophagy is the LC3-II protein, associated with the membranes of autophagosomes and autophagolysosomes [25]. The cytosolic protein LC3-I (microtubule-associated protein 1A/1B-light chain 3) is recruited during autophagy and conjugated with a phosphatidylethanolamine (PE) molecule to form LC3-II. This can then be incorporated into the phagophore membrane and is degraded only after fusion to the lysosome by hydrolases [25]. Its expression is therefore closely correlated with autophagy, and its detection by immunoblot has proved an effective method for monitoring this process [26]. In addition, LC3-II interacts with the p62 protein capable of binding to ubiquitinated proteins or organelles [27]. This interaction ensures selective degradation of components incorporated into the autophagosome. Autophagy is a naturally observed degradation process, which contributes to cell homeostasis by targeting damaged or overexpressed proteins or organelles [24].

**Figure 5:**
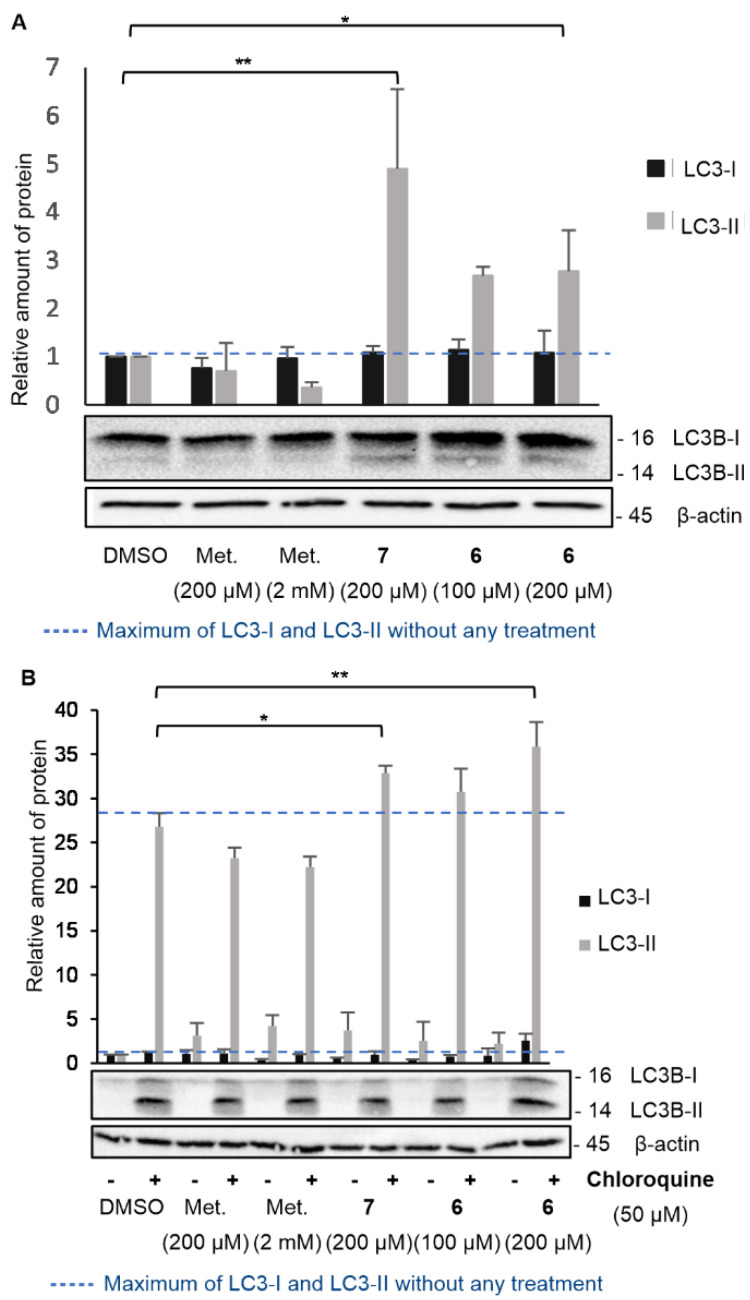
Effect of AUTAC-Biguanide **6** and its controls on LC3-I and LC3-II protein levels. (**A**) Immunoblot for the study of LC3-I and LC3-II protein levels in KP4 after 24 h of treatment and relative quantification of protein levels for metformin (Met.), AUTAC-Hexyl (**7**), and AUTAC-Biguanide (**6**); * *p* ≤ 0.05; ** *p* ≤ 0.001 (ANOVA). (**B**) Effect of AUTAC-Biguanide **6** and its controls on LC3-I and LC3-II protein levels in the absence and presence of chloroquine; immunoblot for the study of LC3-I and LC3-II protein levels in KP4, with and without chloroquine, after 24 h of treatment and relative quantification of protein levels for metformin, **7** and **6**; * *p* ≤ 0.01; ** *p* ≤ 0.001 (ANOVA).

There is therefore a basal level of LC3-II, which may differ depending on the cell line studied or the experimental conditions. To assess the impact of compound **6** on the induction of the autophagy process, LC3-I and LC3-II levels were therefore determined in KP4 after 24 h of different treatments and expressed as a relative quantity compared to the protein level in control-treated cells (DMSO) (Figure 5). According to previous experiments, AUTAC-Biguanide was most effective after a 24 h treatment at 200 µM in KP4, with major disruption of the mitochondrial network. Cells were then treated during the same time with 100 µM (EC_50_) and 200 µM (2 × EC_50_) of compound **6**. To evaluate the impact of the biguanide component on autophagy levels, two different concentrations of metformin were examined: 200 µM, to assess the effect of the biguanide ligand at a concentration equivalent to that of compound **6**, and 2 mM, the concentration at which metformin demonstrates efficacy as an anticancer agent in these cells. For each of these metformin concentrations, it was observed that the level of LC3-II did not increase, indicating that the anticancer properties of metformin are not tied to its ability to induce autophagy (Figure 5A). At a concentration of 200 µM, AUTAC-Hexyl **7** demonstrated an ability to elevate LC3-II levels, underscoring the capacity of the AUTAC ligand to induce autophagy. However, it is important to note that AUTAC lacks anticancer properties and does not affect mitochondrial morphology at higher concentrations. Its participation in the autophagy process does not inherently lead to cytotoxicity, and it does not specifically target mitochondria. On the other hand, AUTAC-Biguanide showed a mild increase in LC3-II levels after treatment at 100 µM and 200 µM. This suggests that it retains the autophagy-inducing properties of the AUTAC ligand, but its conjugation with the biguanide function directs its effect toward mitochondria, potentially leading to mitophagy.

However, when studying the effects of a chemical agent on autophagy, the increase in LC3-II protein levels can result from either enhanced conversion of LC3-I to LC3-II (indicative of autophagy activation) or reduced elimination by the lysosome (indicative of lysosomal degradation inhibition). To determine whether compound **6** serves as an autophagy activator or an inhibitor of lysosomal degradation, the same experiment described above was conducted in the presence of the autophagosome–lysosome fusion inhibitor, chloroquine (Figure 5B). The introduction of chloroquine to the medium led to an increase in LC3-II levels by inhibiting its degradation. A comparison between the medium without chloroquine and with chloroquine helped to determine whether AUTAC-Biguanide exhibited similar properties to this inhibitor. If this were the case, the addition of chloroquine in its presence would have had little impact on LC3-II levels. Immunoblot results revealed a significant overexpression of LC3-II in the presence of chloroquine under all conditions, including when AUTAC-Biguanide was present. Therefore, it can be concluded that AUTAC-Biguanide does not affect the degradation of LC3B.

AUTACs were specifically engineered by Arimoto et al. [15] to induce autophagy in cells through S-guanylation, thereby promoting the degradation of their targets. In our case, the examination of LC3-II levels suggests the activation of upstream autophagy. The incorporation of the biguanide component allows this degradation process to be selectively directed toward mitochondria, consequently inducing mitophagy. However, there is no proof at this time that S-guanylation in cells treated with AUTAC-Biguanide is the reason for the observed mitophagy. Direct evidence could be obtained by employing specific probes that bind to S-guanylated cysteine residues or by quantifying the levels of reactive oxygen species (ROS) and nitric oxide (NO) in the treated cells to ascertain whether the treatment stimulates the synthesis of 8-nitro-cGMP, which may be responsible for S-guanylation. It would also be interesting to ascertain whether there is an increase or decrease in ubiquitinated proteins in cells treated with AUTAC-Biguanide.

## 3. Materials and Methods

**General information:** All chemicals were purchased from Sigma Aldrich (St. Louis, MO, USA), Oakwood Chemicals (Estill, SC, USA), and Combi-Blocks (San Diego, CA, USA) in their highest purity and used without further purification. NMR spectra were recorded on AVANCE II 400, Bruker AVANCE NEO 400, Bruker AVANCE 500 Bruker, and Bruker AVANCE 700 spectrometers (Bruker, Ettlingen, Germany). Purifications of final compounds were performed on a prep LC-MS (Quadrupole) from Waters (Milford, MA, USA). High-resolution mass spectra (HRMS) and LCMS purity analysis were performed on TOF Agilent instruments (Agilent Technologies, Santa Clara, CA, USA) provided by the Regional Mass Spectrometry Centre (Université de Montréal).

**Materials and methods for in vitro experiments**: Absorbance measurements for EC50 determination were performed using a SpectraMax 190 microplates reader from Molecular Devices. Resulting data were analyzed with PRISM software (version 10.3.0) using curve fitting with non-linear regression (log(inhibitor) vs. response—variable slope (four parameters)). Statistical analyses were also performed using the PRISM software, using a one-way ANOVA test. Protein concentrations were determined using a Nanodrop 2000c spectrophotometer from Thermo Scientific (Waltham, MA, USA). A ChemiDoc MP Imaging System from BIO-RAD (Hercules, CA, USA) was used for imaging and analyzing Western blots. Resulting data were treated with Image Lab software (version 6.1).

**Cell Lines:** Human pancreatic normal epithelial (HPNE hTERT) cells were obtained from Dr. M. Ouellette (University of Nebraska Medical Center). IMR90 was obtained from Coriell Institute (I90-79). PANC1 was obtained from ATCC and KP4 from the Massachusetts General Hospital Center for Molecular Therapeutics (CMT), courtesy of Dr. Bardeesy. IMR90, PANC1, and KP4 were cultured in DMEM (319-015-CL, Wisent, Saint-Jean-Baptiste, QC, Canada) supplemented with 1% penicillin/streptomycin (Wisent) and 10% FBS (Wisent or Gibco, Billings, MT, USA). HPNE-hTERT was cultured in media made of 75% DMEM (219-060-XK) and 25% M3 Base (M300F-500, Incell, San Antonio, TX, USA), supplemented with 1 g/L D-glucose (Sigma, Tokyo, Japan), 1.5 g/L sodium bicarbonate (Bioshop, Burlington, ON, Canada), 5% FBS (Wisent), 2 mM L-glutamine (Wisent), 10 ng/mL of human epidermal growth factor (hEGF, Gibco), and phenol red (Sigma). All cell lines tested negative for mycoplasma contamination. No unpublished de novo cell lines were used.

**Cell proliferation assay and determination of the EC_50_:** KP4, PANC1, IMR90, and hTERT-HPNE cells were grown in DMEM (319-015-CL, Wisent) supplemented with 10% fetal bovine serum (FBS, Wisent) and penicillin/streptomycin/L-glutamine (Wisent) at 37% with 5% CO_2_. Cells were seeded at a density of 1 × 10^3^ cells per well for KP4 and PANC1, 4 × 10^3^ for IMR90, and 2 × 10^3^ for hTERT-HPNE in 96 well plates one day prior to treatments with vehicle and compounds for 72 h. The culture medium was removed. Cells were washed twice with cold PBS, and 1% glutaraldehyde was added to the wells for fixation for 10 min. Cells were then washed with water, stained with 0.2% crystal violet for 30 min at room temperature, washed with water, and then dried. The adherent crystal violet was solubilized in 10% acetic acid and the absorbance was read at a wavelength of 590 nm for the calculation of the cell viability.

**Immunoblot analysis:** For the protein degradation assay, cells were seeded at a density of 1 × 10^5^ cells per well in 6-well plates overnight, and then treated with vehicle and compounds for 24 h. The culture medium was removed. Cells were washed twice with cold PBS and lysed with LAEMMLI 2X (BIO-RAD, Hercules, CA, USA, 4% SDS, 20% glycerol, 0.125 M Tris-HCl (pH 6.8)). Equal amounts of protein were separated via sodium dodecyl sulfate-polyacrylamide gel electrophoresis (SDS-PAGE) and transferred to nitrocellulose membranes. Proteins were briefly revealed by ponceau red dye and membranes were washed with PBS-Tween 20 (TBST) before blocking with 5% milk proteins for 1 h. Membranes were washed again with TBST and incubated with the primary antibody overnight at 4 °C. After being washed with TBST three times, the membranes were incubated with the secondary antibody for 1 h. After being washed with TBST three times, membranes were visualized using chemiluminescence using a Western Lightning™ Plus Chemiluminescence Reagent.

Primary Antibodies:

- LC3B Rabbit pAb #2775 (Cell Signaling, Danvers, MA, USA): 1:1000, 5% BSA, 0.1% Tween^®^ 20 in 1X PBS;
- β-Actin (8H10D10) Mouse mAb #3700 (Cell Signaling): 1:10 000, 3% BSA, 0.2% NaN3 in 1X PBS.

Secondary Antibodies:

- Goat Anti-Rabbit IgG (H + L)-HRP Conjugate #1706515 (BioRad, Hercules, CA, USA): 1:1000, 5% non-fat dry milk, 0.1% Tween^®^ 20 in 1X PBS;
- Goat Anti-Mouse IgG (H + L)-HRP Conjugate #1706516 (BioRad): 1:1000, 5% non-fat dry milk, 0.1% Tween^®^ 20 in 1X PBS.

**Cell fluorescence assay:** KP4 cells were seeded at a density of 8 × 10^4^ cells per well on top of microscope cover glasses in 6-well plates overnight. They were then treated with vehicle or the studied compounds for 24 h. The culture medium was refreshed with a solution of the MitoTracker^®^ Deep Red FM (100 nM in DMEM). After 30 min at 37 °C, the medium was removed. Cells were washed twice with PBS and a solution of 4% paraformaldehyde was added into the wells for fixation for 10 min. Cells were washed again three times with 0.1 M glycine in PBS. A drop of mounting medium for fluorescence with DAPI was used to adhere cover glasses to microscope slides.

**Immunofluorescence assay**: KP4 cells were seeded at a density of 8 × 10^4^ cells per well on top of microscope cover glasses in 6-well plates overnight. They were then treated with vehicle or the studied compounds for 24 h. The culture medium was removed, and cells were washed three times with cold PBS. A solution of 4% paraformaldehyde was added into the wells for fixation for 10 min. Cells were washed again three times with 0.1 M glycine in PBS, then incubated with a solution of 3% BSA and 0.2% Triton C-100 in PBS for 5 min at room temperature. Cells were washed again three times with a solution of 3% BSA in PBS, then incubated overnight with the primary antibody TOMM20 (1:100 in PBS, pH 7.4, 3% BSA). Cells were washed three times with 3% BSA in PBS and incubated for 1 h with the secondary antibody AF488 (1:2000 in PBS, pH 7.4, 3% BSA). Cells were finally washed three times with 3% BSA in PBS. A drop of mounting medium for fluorescence with DAPI was used to adhere cover glasses to microscope slides.

Antibodies:

I. Tom20 Antibody (F-10) #sc-17764 (Santa Cruz Biotechnology, Dallas, TX, USA): 1:100, 3% BSA in 1X PBS;
II. Goat anti-Mouse IgG (H + L) Highly Cross-Adsorbed Secondary Antibody, Alexa Fluor™ 488 #A-11029 (ThermoFisher/Invitrogen, Waltham, MA, USA): 1:2000, 3% BSA in 1X PBS.

## 4. Conclusions

AUTAC-Biguanide **6** demonstrates significant antiproliferative properties against cancer cells, leading to mitochondrial perturbations that form a punctate mitochondrial network following treatment at the median effective concentration. Notably, the EC_50_ for AUTAC-Biguanide is significantly lower than that of metformin, underscoring its heightened antiproliferative activity against cancer cells.

Furthermore, AUTAC-Biguanide demonstrates notable selectivity for cancer cells compared to healthy cells. Consequently, this bifunctional molecule proves to be a superior anticancer agent compared to metformin. Moreover, the observed perturbations of the mitochondrial network following treatment with AUTAC-Biguanide serve as compelling evidence that biguanidium salts effectively target these organelles and suggest that the biguanide function could be considered a mitochondria-targeting agent. 

In contrast to the mito-AUTAC previously reported that improved cell viability [15], we present new biguanide-based AUTACs that are toxic to cancer cells and thus hold potential for further developments in anticancer therapies. The action of AUTACs may depend on the presence of 8-nitro-cGMP, a metabolite produced from cGMP by the action of nitric oxide (NO) and ROS. Pancreatic cancer cells bearing KRAS mutations exhibit high levels of ROS and NO, suggesting why biguanide AUTACs may be toxic to these cells, which likely have elevated levels of 8-nitro-cGMP. Further in vivo studies are needed to support this hypothesis.

The synthesis of this biguanide derivative, capable of inducing autophagy, has therefore paved the way for the development of more potent anticancer agents.

## Figures and Tables

**Table 1:** EC_50_ and selectivity index (SI) of AUTAC-Biguanide and its controls, determined after 72 h of treatment in different cell lines (mean ± SD).

Entry	EC_50_ (µM)				
	KP4	PANC1	IMR90	hTERT-HPNE	SI
Met. ^a^	1.49 ± 0.55	3.13 ± 1.41	>20	>20	>13
**5**	>5 *	>5 *	>5 *	3.51 ± 1.45	-
**6**	0.10 ± 0.03	0.15 ± 0.03	>1 *	> 1 *	>10
**7**	>1 *	>1 *	>1 *	0.11 ± 0.02	-

See section “Material and Methods”. * Could not be determined due to solubility issues. ^a^ Met. = Metformin.

## Data Availability

The original contributions presented in the study are included in the article/Supplementary Material, further inquiries can be directed to the corresponding author.

## Supplementary Materials

The following supporting information can be downloaded at: https://www.mdpi.com/article/10.3390/molecules29163773/s1, Synthetic procedures, characterization of the compounds, and confocal microscopy images of KP4 cells.
