# Supplementary material for: Gaining Insight into Mitochondrial Targeting: AUTAC-Biguanide as an Anticancer Agent
Source: Molecules. 2024 Aug 9;29(16):3773. doi: 10.3390/molecules29163773 (PMC11357661; doi:10.3390/molecules29163773)
Supplement: Supplementary file 1 [file molecules-29-03773-s001.zip › molecules-3067939-supplementary.pdf]

Supporting Information for

Gaining Insight into Mitochondrial Targeting: AUTAC-Biguanide as an Enhanced Anticancer Agent

Julie Vatté,[1] Véronique Bourdeau,[2] Gérardo Ferbeyre,[2,3] and Andreea R. Schmitzer*[1]

Contents of Supporting Information

1. **Synthetic procedures of compounds 1 to 7……………………………………………………….. S2-7**
2. **Characterization data of products 1 to 7……………………………………………………… S7-21**
3. **Confocal microscopy images of KP4 cells…………………………………………………………S22**

Synthetic procedures of compounds 1 to 7

6-Aminohexylbiguanide hydrochloride (1)

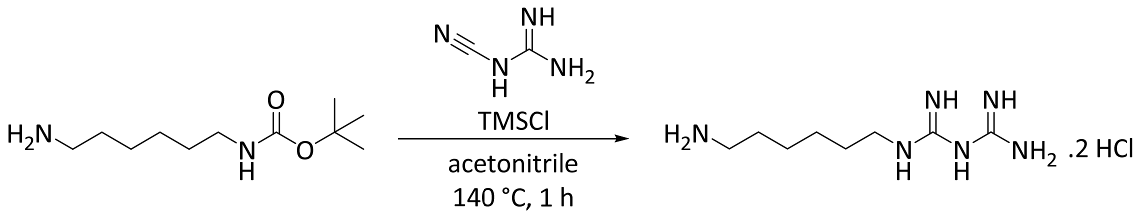

tert-Butyl (6-aminohexyl)carbamate (1.22 g, 5.64 mmol) was dissolved in 15 mL of acetonitrile. Dicyandiamide (474 mg, 5.64 mmol) and chlorotrimethylsilane (1.43 mL, 11.30 mmol) were added and the mixture was heated to 150 °C in a sealed tube for 1 h. The solution was cooled to room temperature and 1.4 mL of HCl in dioxane (4 M) was added. The suspension was stirred for 15 min. The precipitate was filtered and washed with ethyl acetate to afford 1.20 g of a white powder (Yield 78 %).

^{1}H NMR (500 MHz, DMSO-*d$_6$*) δ 9.50 (s, 1H), 9.09 (s, 2H), 8.51 (s, 4H), 8.16 (s, 2H), 7.71 (s, 1H), 3.25 (s, 2H), 2.74 (q, *J* = 6.8 Hz, 2H), 1.58 – 1.52 (m, 4H), 1.35 – 1.32 (m, 4H)

^{13}C NMR (126 MHz, DMSO-*d$_6$*) δ 155.5, 152.4, 39.0, 27.2, 26.1, 26.0, 25.9

HRMS: m/z $[M + H]^+$ calcd for $C_8H_{20}N_6$, 201.18222; found, 201.18152, $[M+Na]^+$ calcd 223.16417; found, 223.16232

6-Chloro-9-(4-fluorobenzyl)-9*H*-purin-2-amine (2)

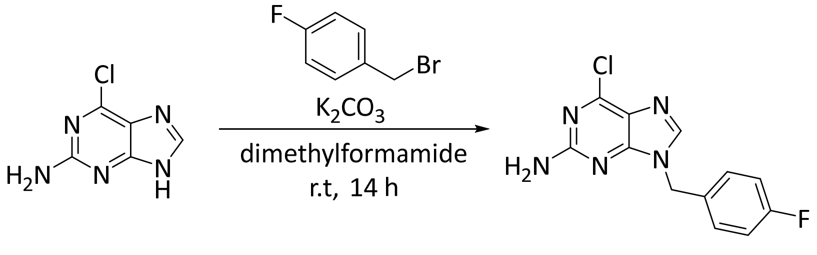

2-Amino-6-chloropurine (200 mg, 1.18 mmol) was dissolved in dimethylformamide (2.00 mL) and potassium Carbonate anhydrous (196 mg, 1.42 mmol) was added. The solution was stirred under

N_2 atmosphere for 1 h. 4-fluorobenzyl bromide (132 µL, 1.06 mmol) was added and the reaction mixture was stirred for 12 h under N_2 atmosphere at room temperature. The mixture was then concentrated under vacuum and purified by chromatography on silica gel with eluent 0 to 5 % methanol in dichloromethane to afford 262 mg of the product (Yield 89 %).

^{1}H NMR (500 MHz, DMSO-*d$_6$*) δ 8.23 (s, 1H), 7.38 – 7.30 (m, 2H), 7.23 – 7.15 (m, 2H), 6.96 (s, 2H), 5.28 (s, 2H)

^{13}C NMR (126 MHz, DMSO-*d$_6$*) δ 162.6, 159.9, 154.0, 149.5, 143.1, 132.9, 129.5, 129.4, 123.3, 115.6, 115.5, 45.4

<u>2-Amino-9-(4-fluorobenzyl)-1,9-dihydro-6*H*-purin-6-one (3)</u>

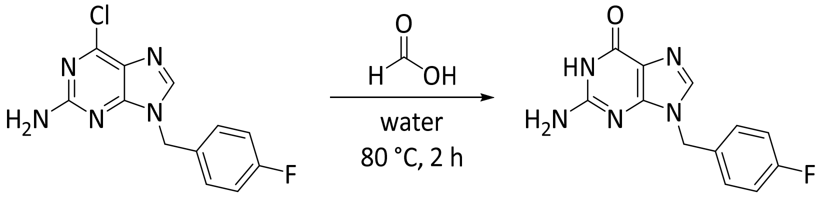

6-Chloro-9-(4-fluorobenzyl)-9H-purin-2-amine (92.0 mg, 331 µmol) was dissolved in 80 % formic acid solution (2.5 mL) and heated to 80 °C for 2 h. The mixture was concentrated under reduced pressure to afford 100 mg of the title compound as a white formate salt (Quantitative yield).

^{1}H NMR (400 MHz, DMSO-*d$_6$*) δ 11.95 (s, 1H), 8.85 (s, 1H), 8.15 (s, 1H), 7.49 (dd, *J* = 8.4, 5.3 Hz, 2H), 7.41 (s, 2H), 7.21 (t, *J* = 8.7 Hz, 2H), 5.50 (s, 2H)

^{13}C NMR (126 MHz, DMSO-*d$_6$*) δ 163.0, 162.9, 160.9, 154.0, 153.2, 151.4, 140.3, 132.2, 130.3, 130.2, 115.6, 115.5, 107.1, 49.3, 40.0, 39.9, 39.7, 39.5, 39.4, 39.2, 39.0

<u>2-Amino-8-bromo-9-(4-fluorobenzyl)-1,9-dihydro-6*H*-purin-6-one (4)</u>

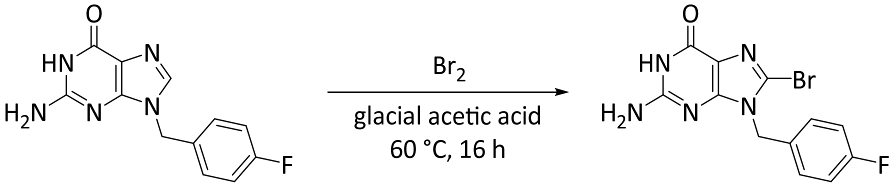

2-Amino-9-(4-fluorobenzyl)-1,9-dihydro-6*H*-purin-6-one (237 mg, 914 µmol) was dissolved in glacial acetic acid (16.6 mL) and stirred for 2 minutes. Bromine water (186 µL, 3.61 mmol) was added and the mixture was heated to 60 °C for 16 h. Water was added to the cooled solution and the precipitate was filtered off and dried under reduced pressure to afford 136 mg of a mixture of 2 isomers as a white powder (Yield isomer 1 : 22 % ; isomer 2 : 22 %).

^{1}H NMR (400 MHz, DMSO-*d$_6$*) δ 10.76 (s, 1H), 10.73 (s, 1H), 7.25 (td, *J* = 10.3, 9.4, 5.4 Hz, 4H), 7.18 (t, *J* = 8.7 Hz, 4H), 6.63 (s, 4H), 5.15 (m, 4H)

^{13}C NMR (101 MHz, DMSO-*d$_6$*) δ 162.8, 160.4, 155.6, 155.6, 154.2, 154.2, 152.6, 152.2, 132.2, 132.1, 131.6, 129.1, 129.1, 129.0, 128.9, 120.7, 116.8, 115.7, 115.7, 115.5, 115.5, 115.1, 45.6, 44.8

***N*-Acetyl-S-(2-amino-9-(4-fluorobenzyl)-6-oxo-6,9-dihydro-1*H*-purin-8-yl)cysteine (5)**

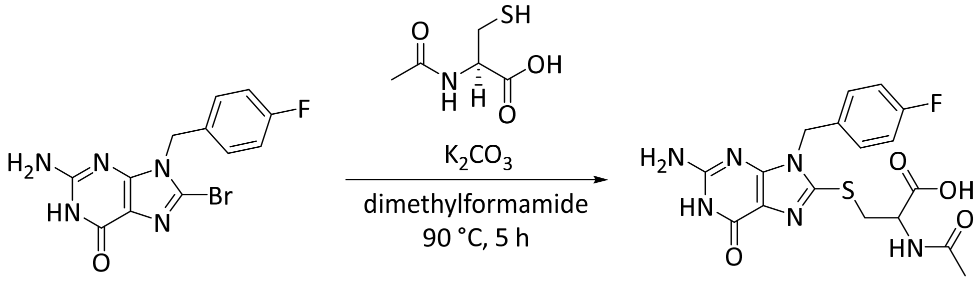

2-Amino-8-bromo-9-(4-fluorobenzyl)-1,9-dihydro-6*H*-purine-6-one (1.00 g, 2.96 mmol) was dissolved in dimethylformamide (15.0 mL). Potassium carbonate anhydrous (4.78 g, 34.6 mmol) and *N*-acetyl-L-cysteine (1.93 g, 11.8 mmol) were added and the mixture was heated to 90 °C for 5 h. The reaction mixture was poured into 20 mL of cold water and concentrated HCl was added until pH 4 was reached. The precipitate was filtered off, washed with water and dried under reduced pressure to obtain 1.00 g of a white powder (Yield 80 %).

^{1}H NMR (500 MHz, DMSO-d_6) δ 12.90 (s, 1H), 10.65 (s, 1H), 8.52 (d, *J* = 7.9 Hz, 1H), 7.28 – 7.07 (m, 4H), 6.53 (s, 2H), 5.08 (d, *J* = 4.8 Hz, 2H), 4.47 (ddd, *J* = 9.0, 7.8, 4.6 Hz, 1H), 3.57 (dd, *J* = 13.6, 4.6 Hz, 1H), 3.28 (dd, *J* = 13.5, 8.9 Hz, 1H), 1.83 (s, 3H)

^{13}C NMR (126 MHz, DMSO-d_6) δ 172.2, 169.8, 163.0, 161.0, 156.1, 154.2, 153.3, 142.3, 133.0, 129.5, 129.4, 117.1, 116.1, 115.9, 52.2, 45.1, 34.3, 22.8

2-Acetamido-3-((2-amino-9-(4-fluorobenzyl)-6-oxo-6,9-dihydro-1*H*-purin-8-yl)thio)-*N*-(6-(3-carbamimidoylguanidino)hexyl)propanamide formate (6)

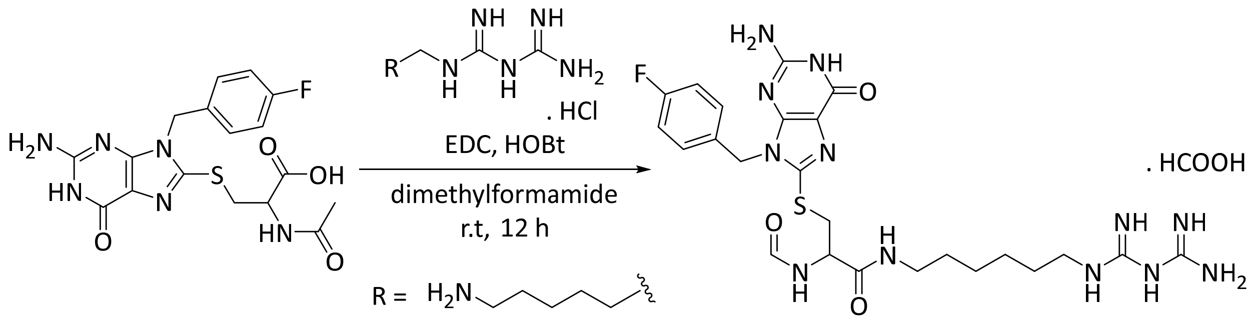

N-Acetyl-S-(2-amino-9-(4-fluorobenzyl)-6-oxo-6,9-dihydro-1*H*-purin-8-yl)cysteine (279 mg, 664 µmol), *N*-(3-dimethylaminopropyl)-*N'*-ethylcarbodiimide (280 µL, 1.58 mmol) and 1-hydroxytriazole hydrate (179 mg, 1.33 mmol) were dissolved in dimethylformamide (22.0 mL) and triethylamine (370 µL, 2.65 mmol) was added. The mixture was stirred for 10 minutes then aminohexylbiguanide (335 mg, 1.41 mmol) was added and the solution was stirred at room temperature for 12 h. Dichloromethane was added to the reaction mixture and the precipitate was filtered off and purified by HPLC prep. The resulting product was poured into water and lyophilised to afford 52 mg of a white solid (Yield 12 %).

^{1}H NMR (400 MHz, DMSO-d_6) δ 11.40 (s, 1H), 8.54 – 8.38 (m, 2H), 8.05 (t, *J* = 5.7 Hz, 1H), 7.67 (s, 1H), 7.23 – 7.13 (m, 4H), 6.93 (d, *J* = 20.8 Hz, 7H), 5.08 (s, 2H), 4.45 (td, *J* = 8.3, 5.1 Hz, 1H), 3.43 (dd, *J* = 13.3, 5.1 Hz, 2H), 3.26 (dd, *J* = 13.3, 8.4 Hz, 2H), 3.05 – 3.00 (m, 2H), 1.84 (s, 3H), 1.39 (dt, *J* = 13.3, 6.5 Hz, 4H), 1.23 (d, *J* = 4.9 Hz, 4H)

^{13}C NMR (101 MHz, DMSO-*d*$_6$) δ 169.4, 166.9, 156.3, 154.1, 152.6, 141.1, 134.2, 129.0, 128.9, 118.8, 115.6, 115.4, 53.5, 45.7, 37.2, 35.9, 28.8, 25.9, 22.6

LC-MS (ESI) : (90 % H_2O + 0.1 % FA to 95 % ACN + 0.1 % FA in 15 min, +ESI TIC), t_R = 5.12 min, 98 % purity

HRMS : m/z $[M+H]^+$ calcd for $C_{25}H_{35}FN_{12}O_3S$, 603.27326; found 603.27418

2-Acetamido-3-((2-amino-9-(4-fluorobenzyl)-6-oxo-6,9-dihydro-1*H*-purin-8-yl)thio)-*N*-hexylpropanamide (7)

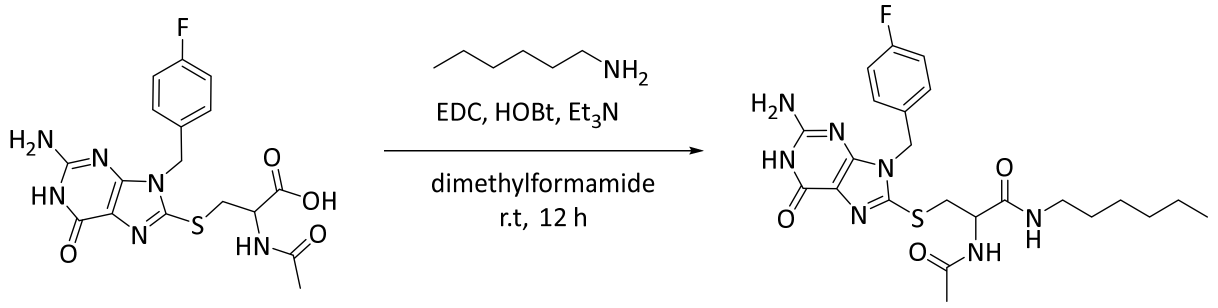

N-Acetyl-S-(2-amino-9-(4-fluorobenzyl)-6-oxo-6,9-dihydro-1*H*-purin-8-yl)cysteine (500 mg, 1.19 mmol), *N*-(3-Dimethylaminopropyl)-*N*′-ethylcarbodiimide (421 µL, 2.38 mmol) and 1-hydroxytriazole hydrate (321 mg, 2.38 mmol) were dissolved in dimethylformamide (39 mL) and triethylamine (332 µL, 2.38 mmol) was added. The mixture was stirred for 10 minutes then hexylamine (189 µL, 1.43 mmol) was added and the solution was stirred at room temperature for 12 h. Water was added to the reaction mixture and the precipitate was filtered off and washed with acetonitrile to afford 343 mg of a white solid (Yield 57 %).

^{1}H NMR (400 MHz, DMSO-*d*$_6$) δ 10.67 (s, 1H), 8.40 (d, *J* = 8.0 Hz, 1H), 8.01 (t, *J* = 5.7 Hz, 1H), 7.24 – 7.12 (m, 4H), 6.55 (s, 2H), 5.09 (s, 2H), 4.47 (td, *J* = 8.0, 5.5 Hz, 1H), 3.42 (dd, *J* = 13.2, 5.5 Hz, 1H), 3.32 – 3.25 (m, 1H), 3.10 – 2.95 (m, 2H), 1.85 (s, 3H), 1.36 (t, *J* = 7.0 Hz, 2H), 1.27 – 1.16 (m, 6H), 0.91 – 0.79 (m, 3H)

^{13}C NMR (101 MHz, DMSO-d_6) δ 169.8, 163.2, 160.8, 156.1, 154.2, 153.2, 142.6, 133.1, 130.1, 130.0, 129.5, 129.4, 117.1, 116.1, 115.9, 115.7, 115.5, 52.8, 45.1, 39.1, 35.3, 31.4, 29.3, 26.4, 23.0, 22.5, 14.4

LC-MS (ESI) : (90 % H_2O + 0.1 % FA to 90 % ACN + 0.1 % FA in 15 min, +ESI TIC), t_R = 6.89 min, 100 % purity

HRMS : m/z $[M+H]^+$ calcd for $C_{23}H_{30}FN_7O_3S$, 504.21876; found 504.21678

1. Characterization data of products 1 to 7

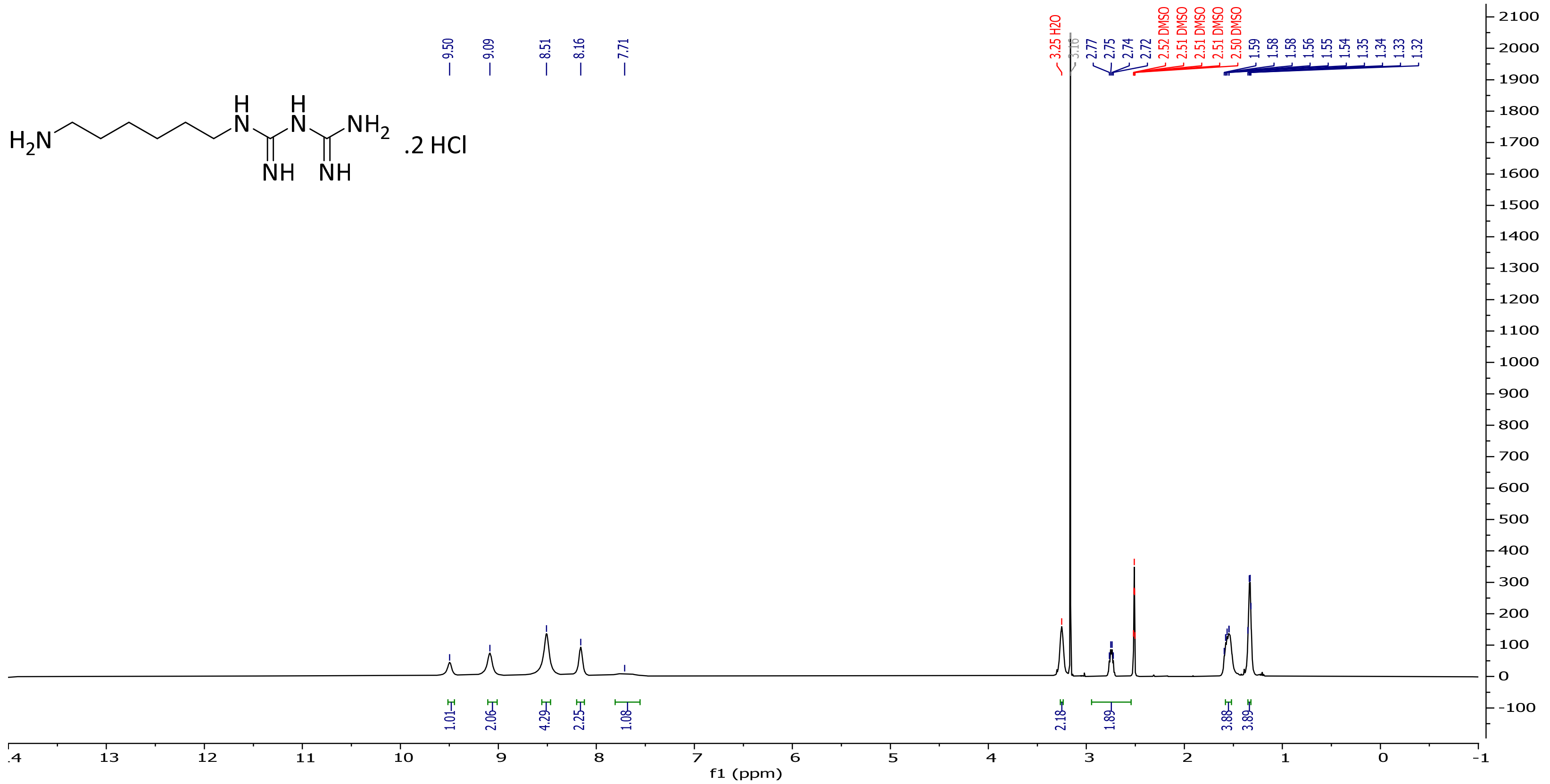

Figure S1. ^{1}H NMR (500 MHz) spectrum of 6-aminohexylbiguanide hydrochloride (1) in DMSO-d_6

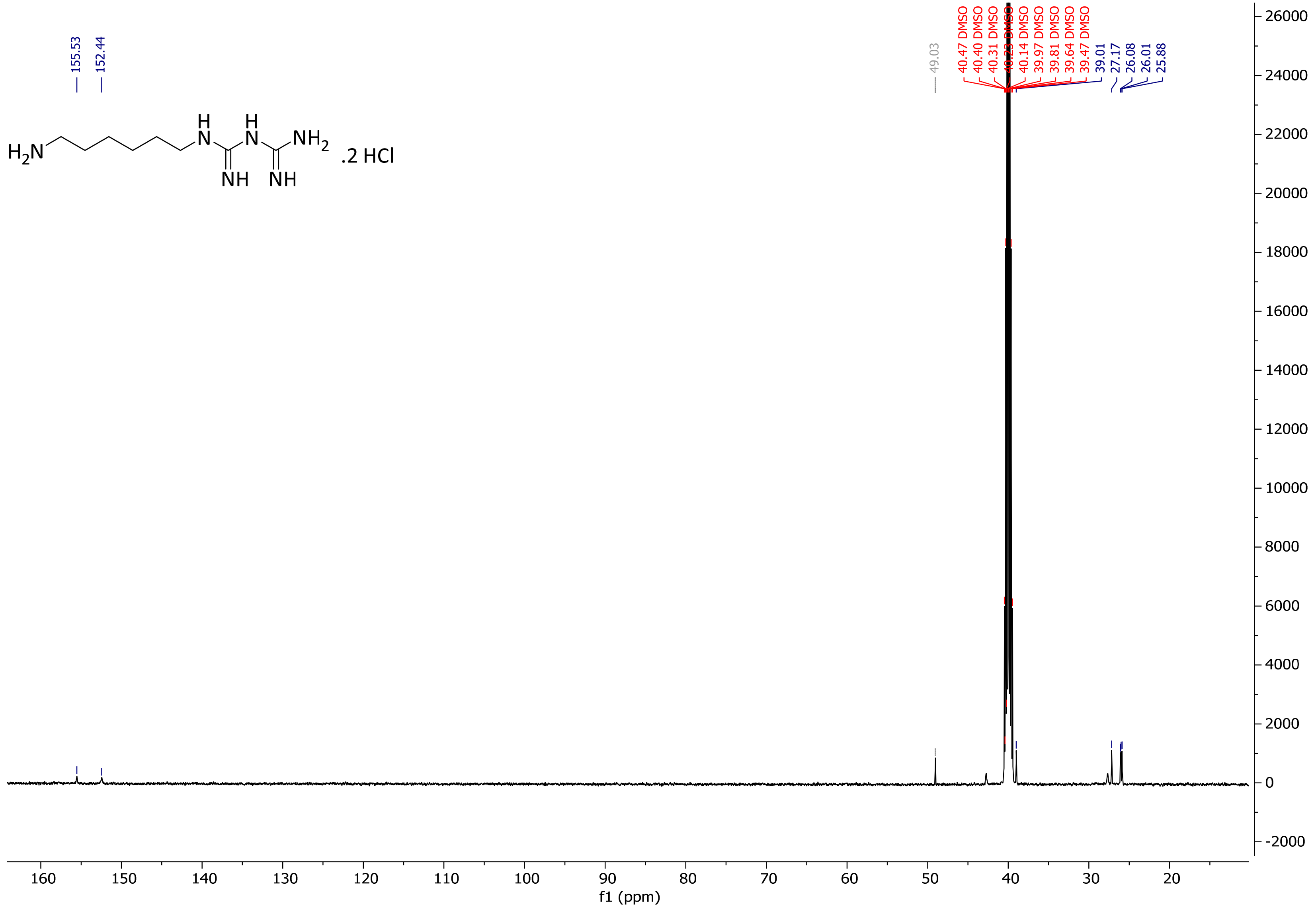

Figure S2. ^{13}C NMR (126 MHz) spectrum of 6-aminohexylbiguanide hydrochloride (1) in DMSO-d_6

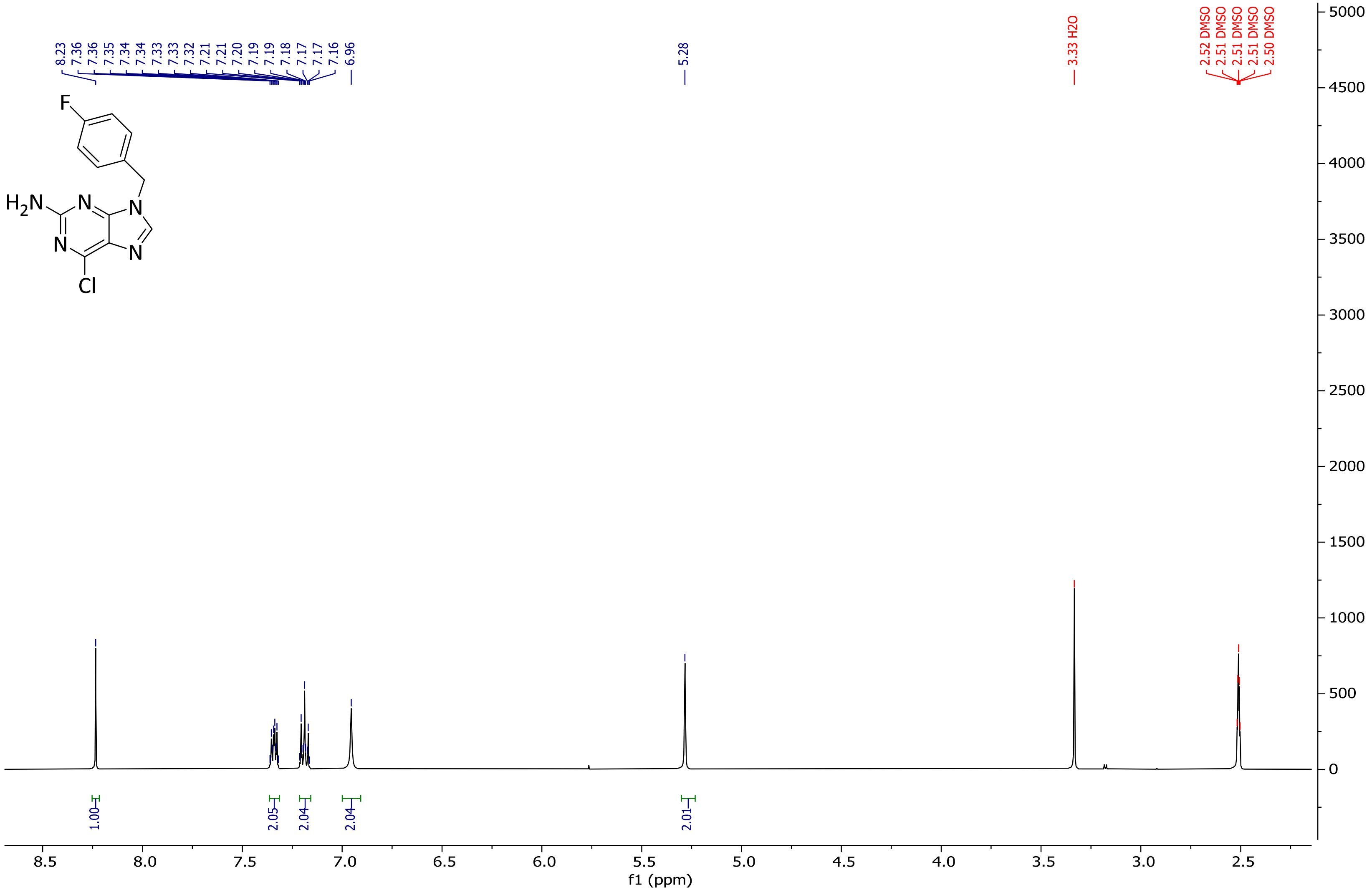

Figure S3. ^{1}H NMR (500 MHz) spectrum of 6-chloro-9-(4-fluorobenzyl)-9H-purin-2-amine (2) in DMSO-d_6

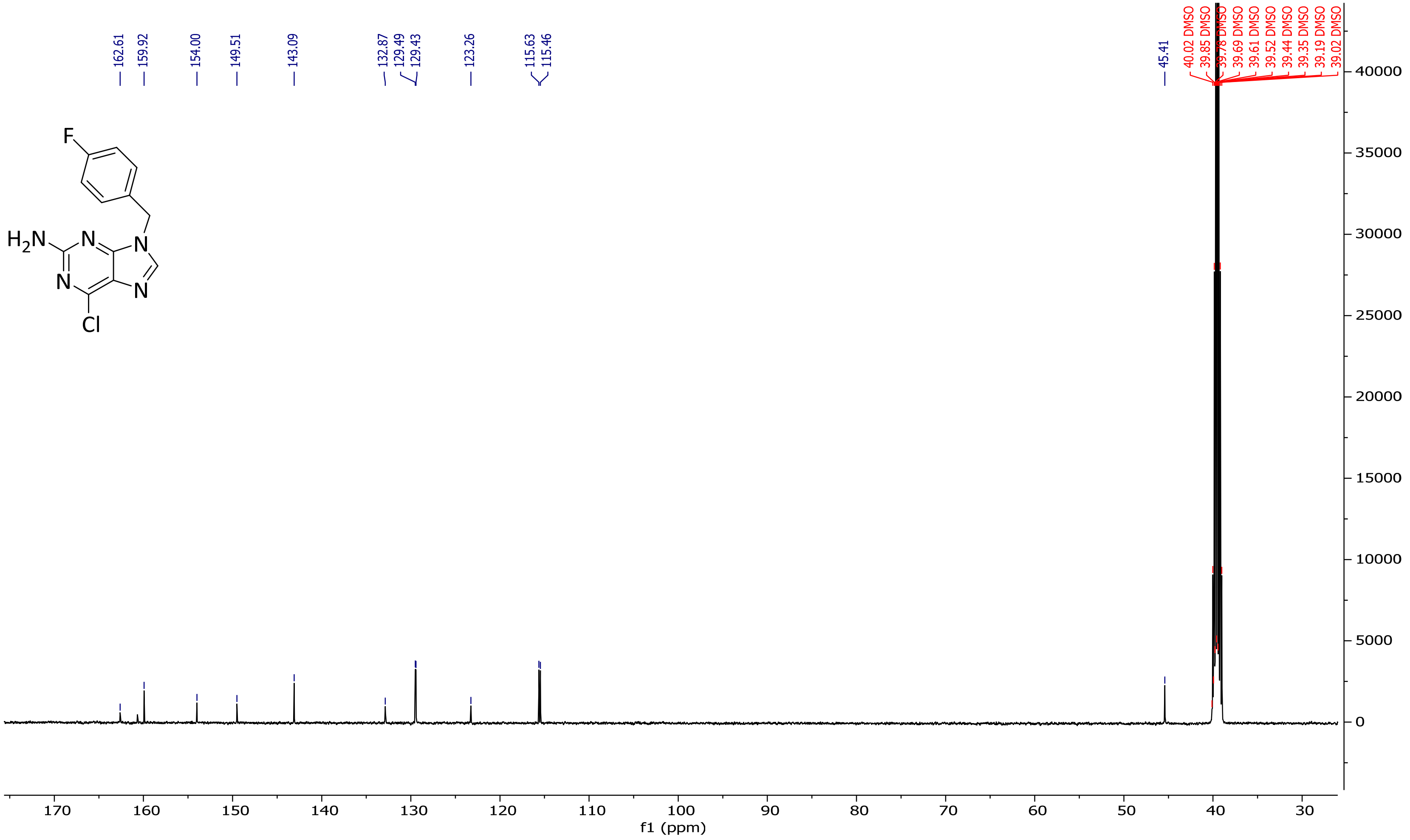

Figure S4. ^{13}C NMR (126 MHz) spectrum of 6-chloro-9-(4-fluorobenzyl)-9H-purin-2-amine (2) in DMSO-d_6

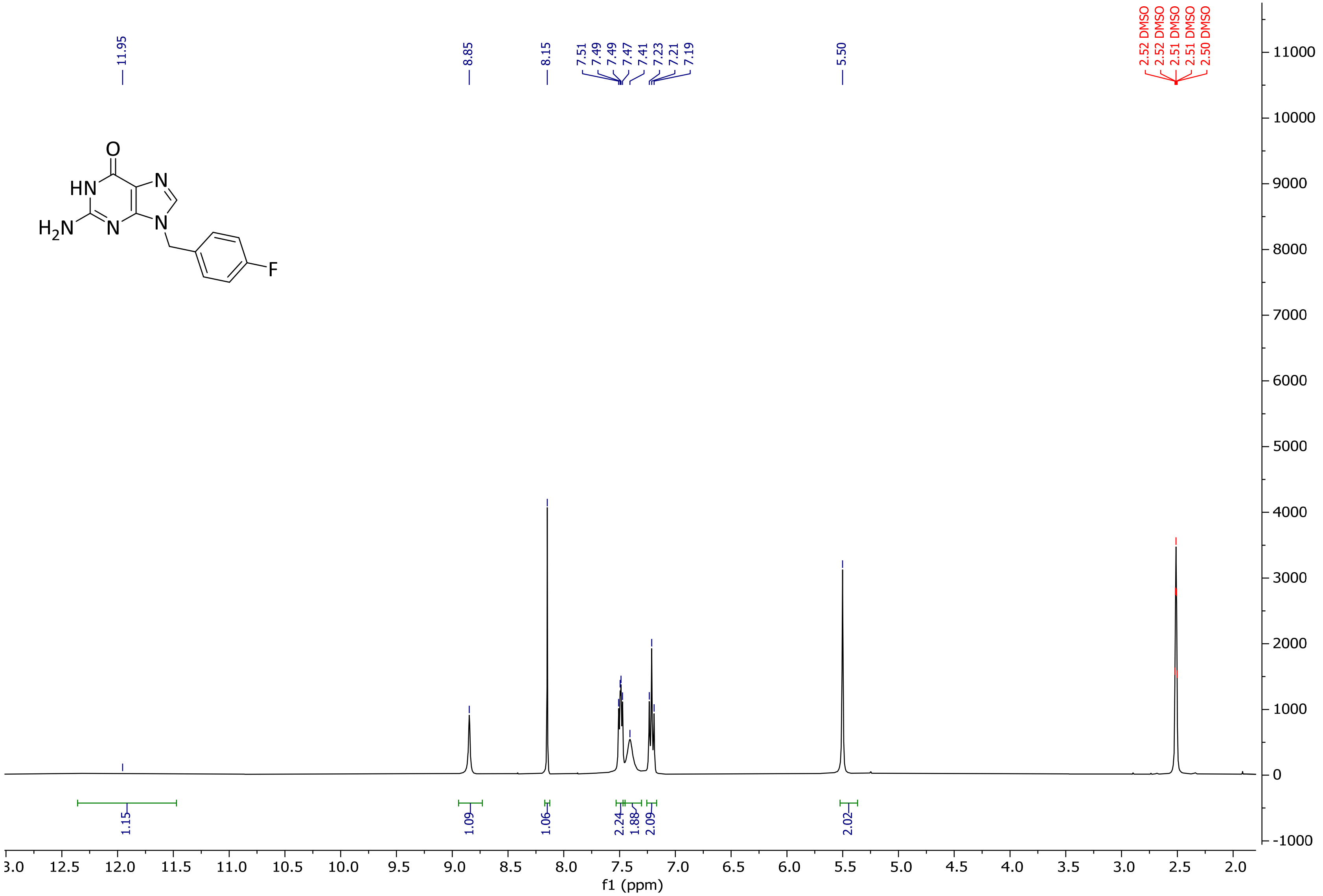

Figure S5. ^{1}H NMR (400 MHz) spectrum of 2-amino-9-(4-fluorobenzyl)-1,9-dihydro-6H-purin-6-one (3) in DMSO-d_6

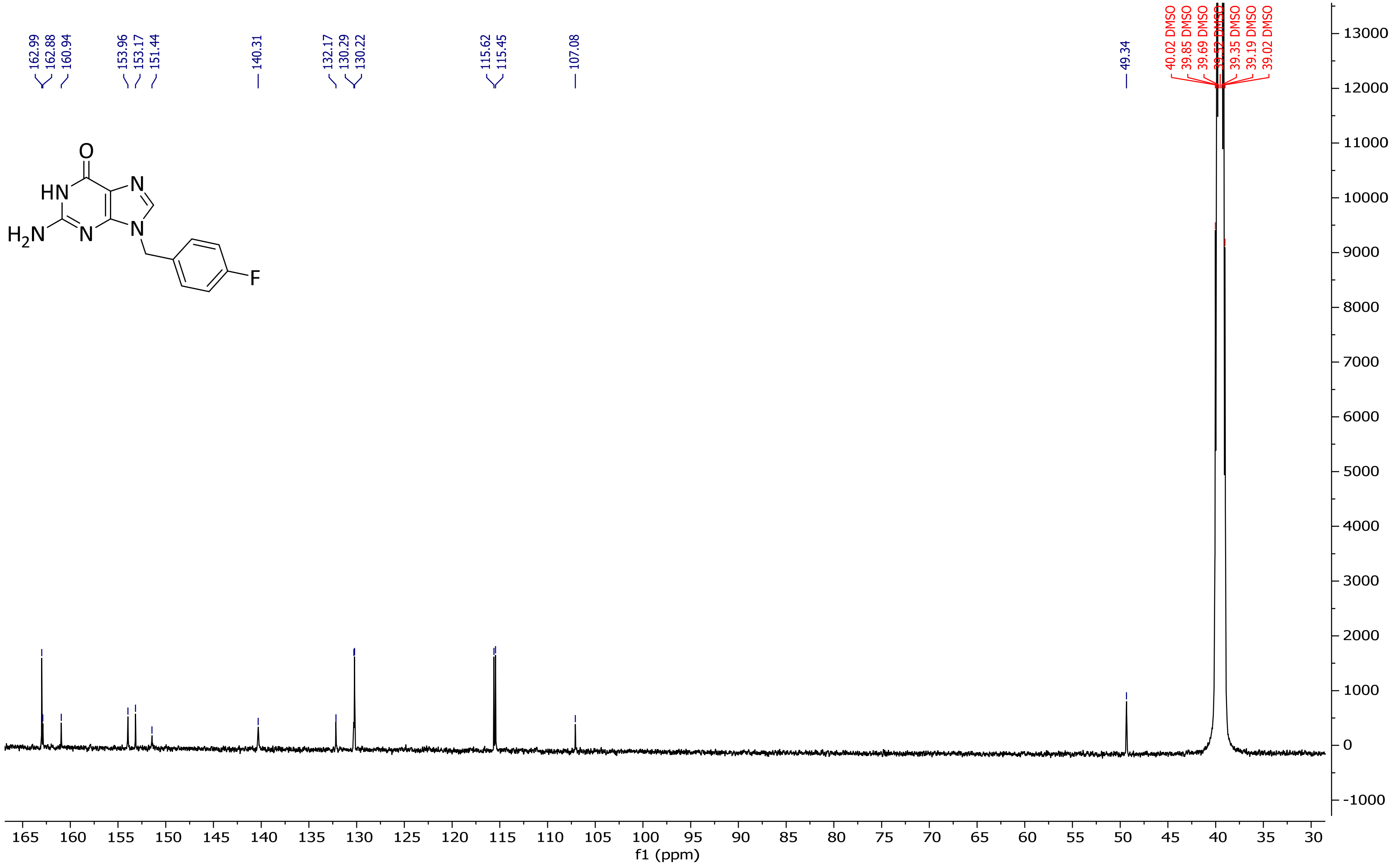

Figure S6. ^{13}C NMR (101 MHz) spectrum of 2-amino-9-(4-fluorobenzyl)-1,9-dihydro-6H-purin-6-one (3) in DMSO-d_6

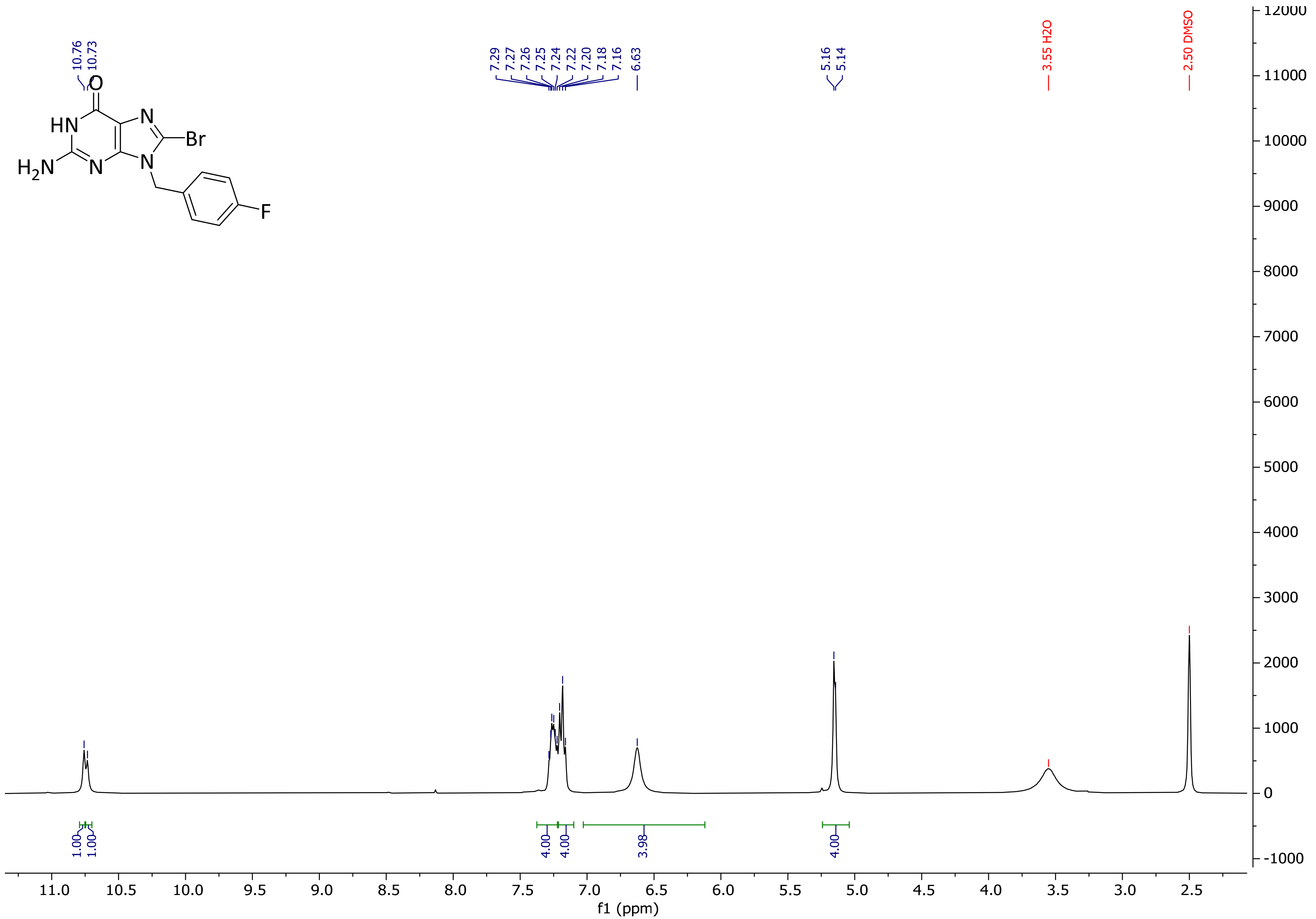

Figure S7. ^{1}H NMR (400 MHz) spectrum of 2-amino-8-bromo-9-(4-fluorobenzyl)-1,9-dihydro-6H-purin-6-one (4) in DMSO-d_6

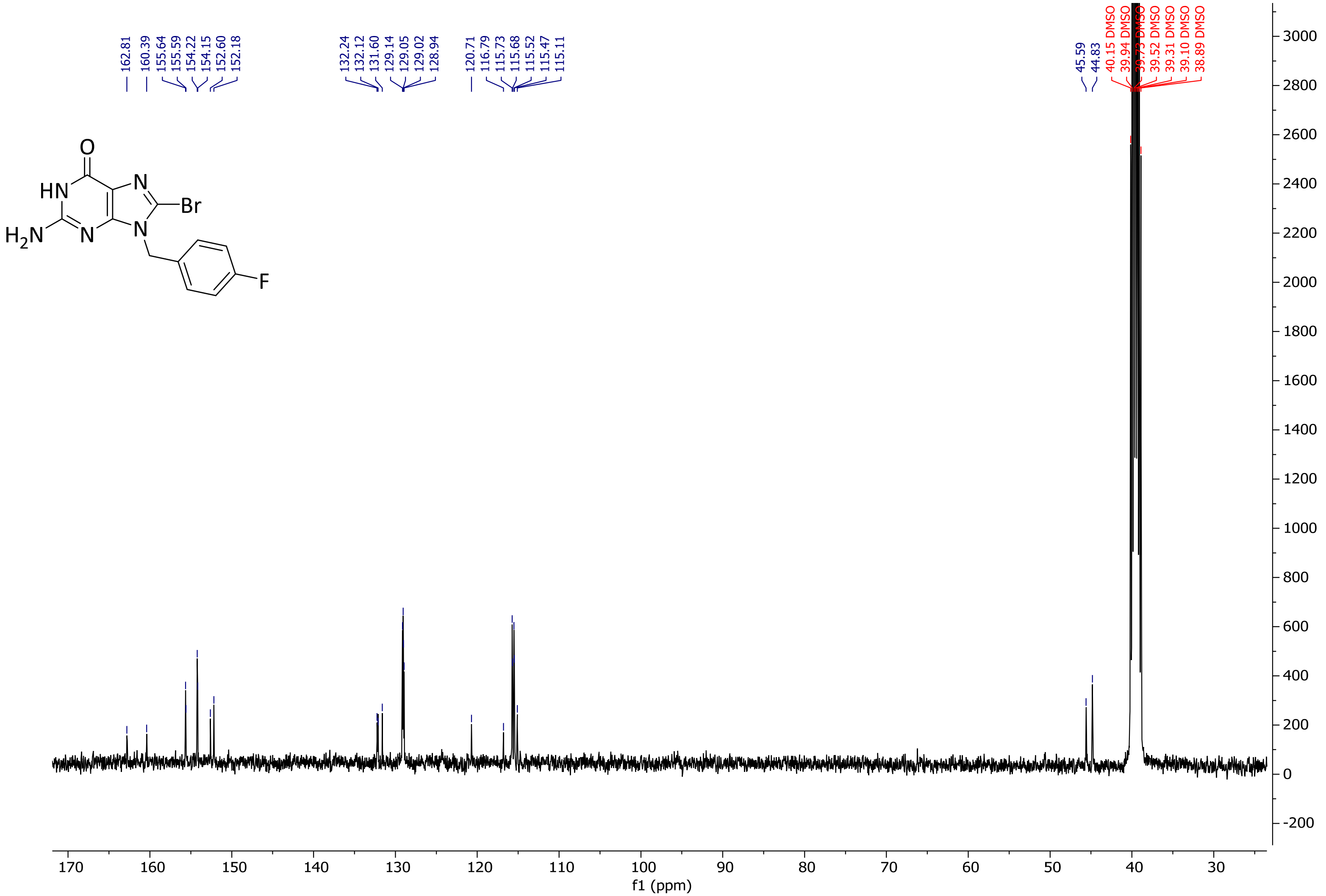

Figure S8. ^{13}C NMR (101 MHz) spectrum of 2-amino-8-bromo-9-(4-fluorobenzyl)-1,9-dihydro-6H-purin-6-one (4) in DMSO-*d*$_6$

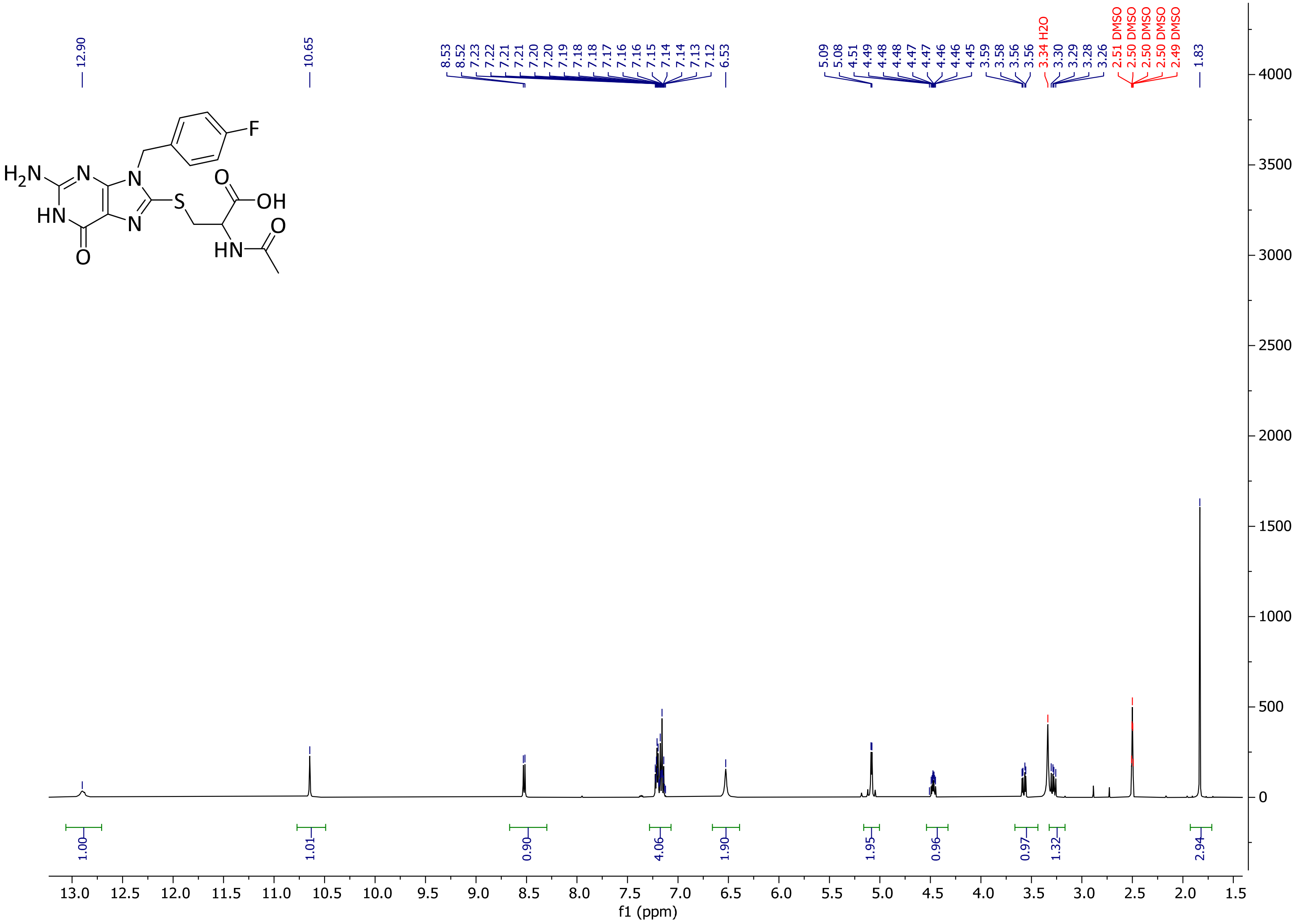

Figure S9. ^{1}H NMR (500 MHz) spectrum of *N*-acetyl-S-(2-amino-9-(4-fluorobenzyl)-6-oxo-6,9-dihydro-1H-purin-8-yl)cysteine (5) in DMSO-d_6

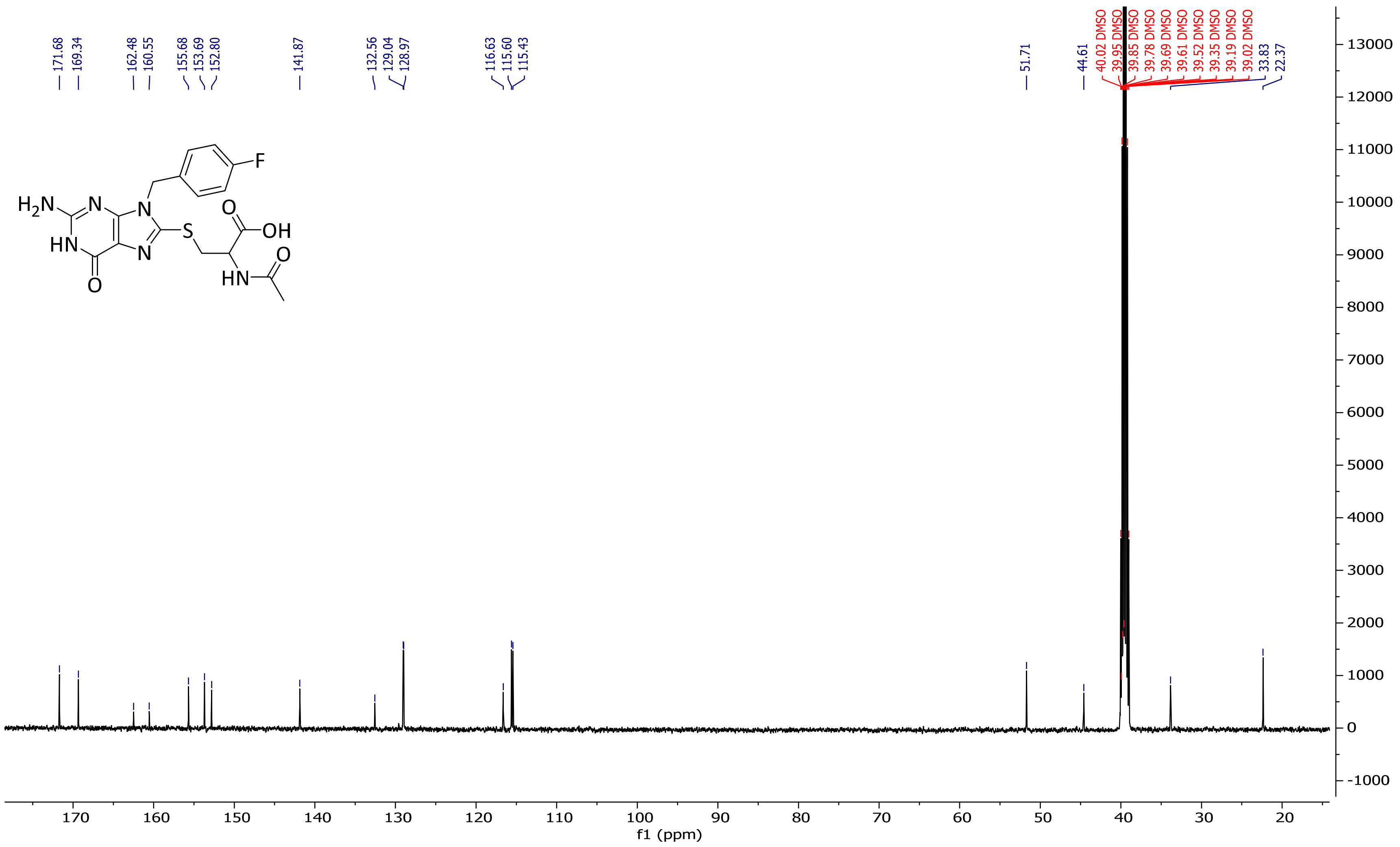

Figure S10. ^{13}C NMR (126 MHz) spectrum of *N*-acetyl-S-(2-amino-9-(4-fluorobenzyl)-6-oxo-6,9-dihydro-1H-purin-8-yl)cysteine (5) in DMSO-d_6

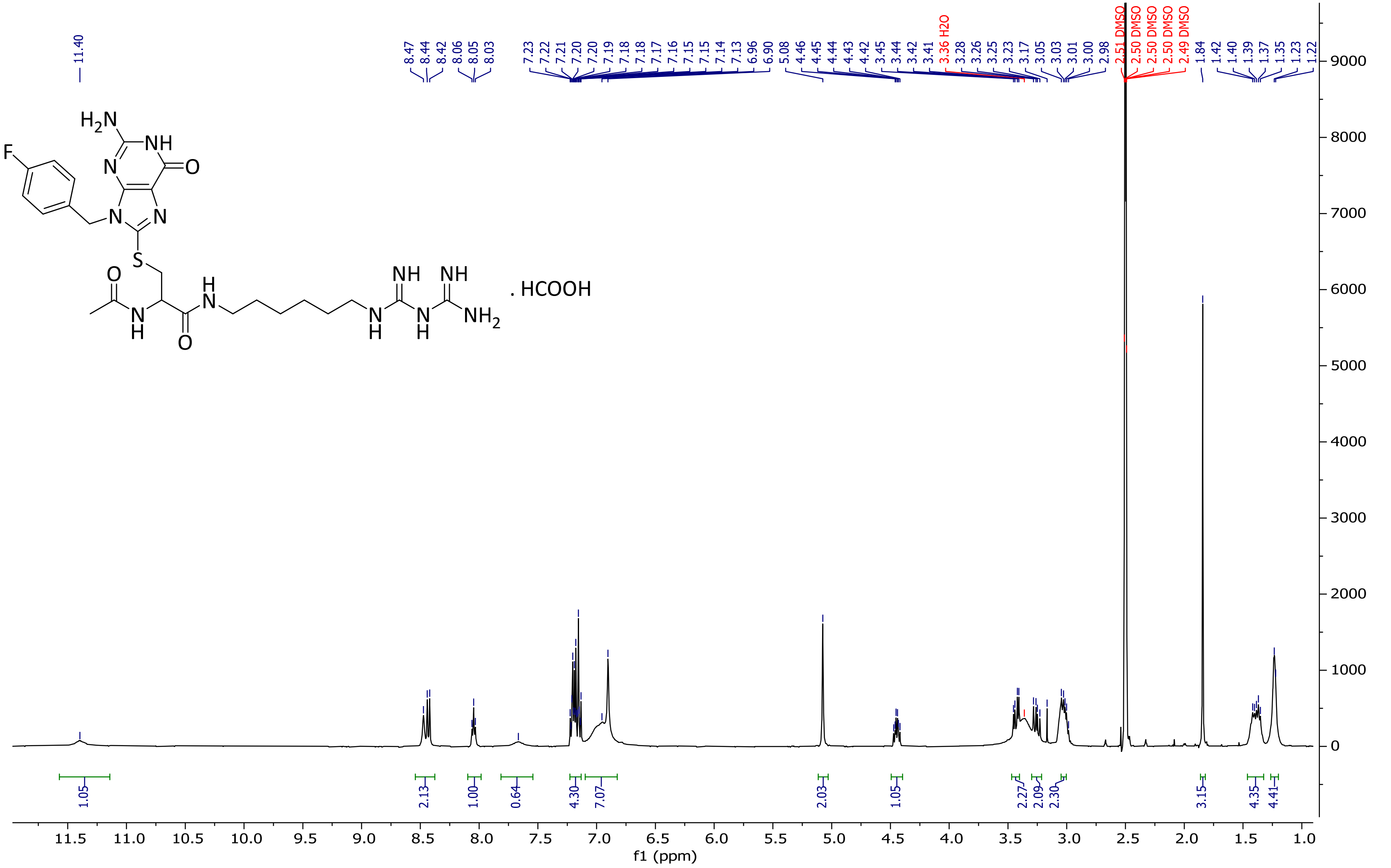

Figure S11. ^{1}H NMR (400 MHz) spectrum of 2-acetamido-3-((2-amino-9-(4-fluorobenzyl)-6-oxo-6,9-dihydro-1H-purin-8-yl)thio)-*N*-(6-(3-carbamimidoylguanidino)hexyl) propanamide formate (6) in DMSO-d_6

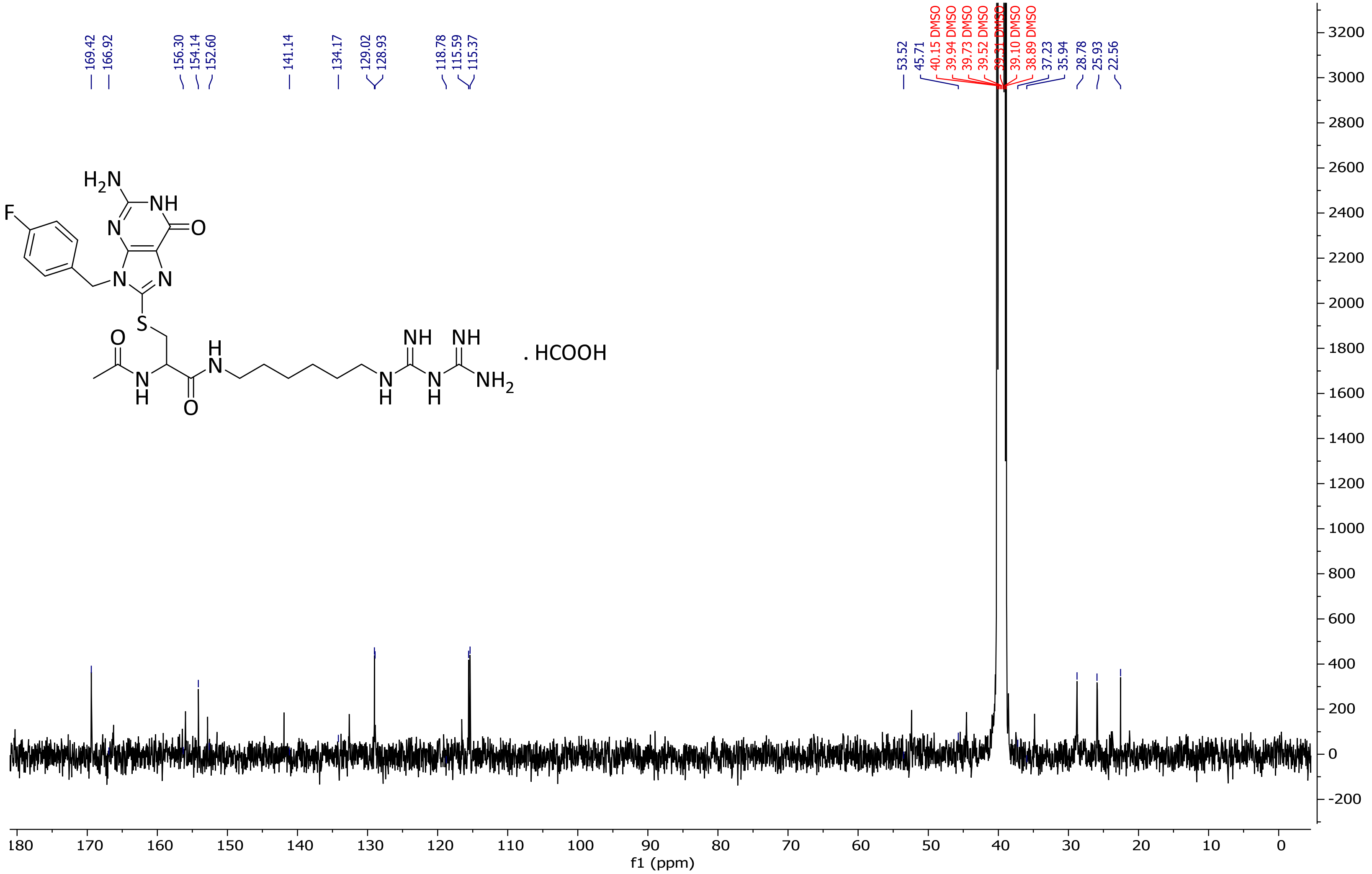

Figure S12. ^{13}C NMR (101 MHz) spectrum of 2-acetamido-3-((2-amino-9-(4-fluorobenzyl)-6-oxo-6,9-dihydro-1H-purin-8-yl)thio)-*N*-(6-(3-carbamimidoylguanidino)hexyl) propanamide formate (6) in DMSO-d_6

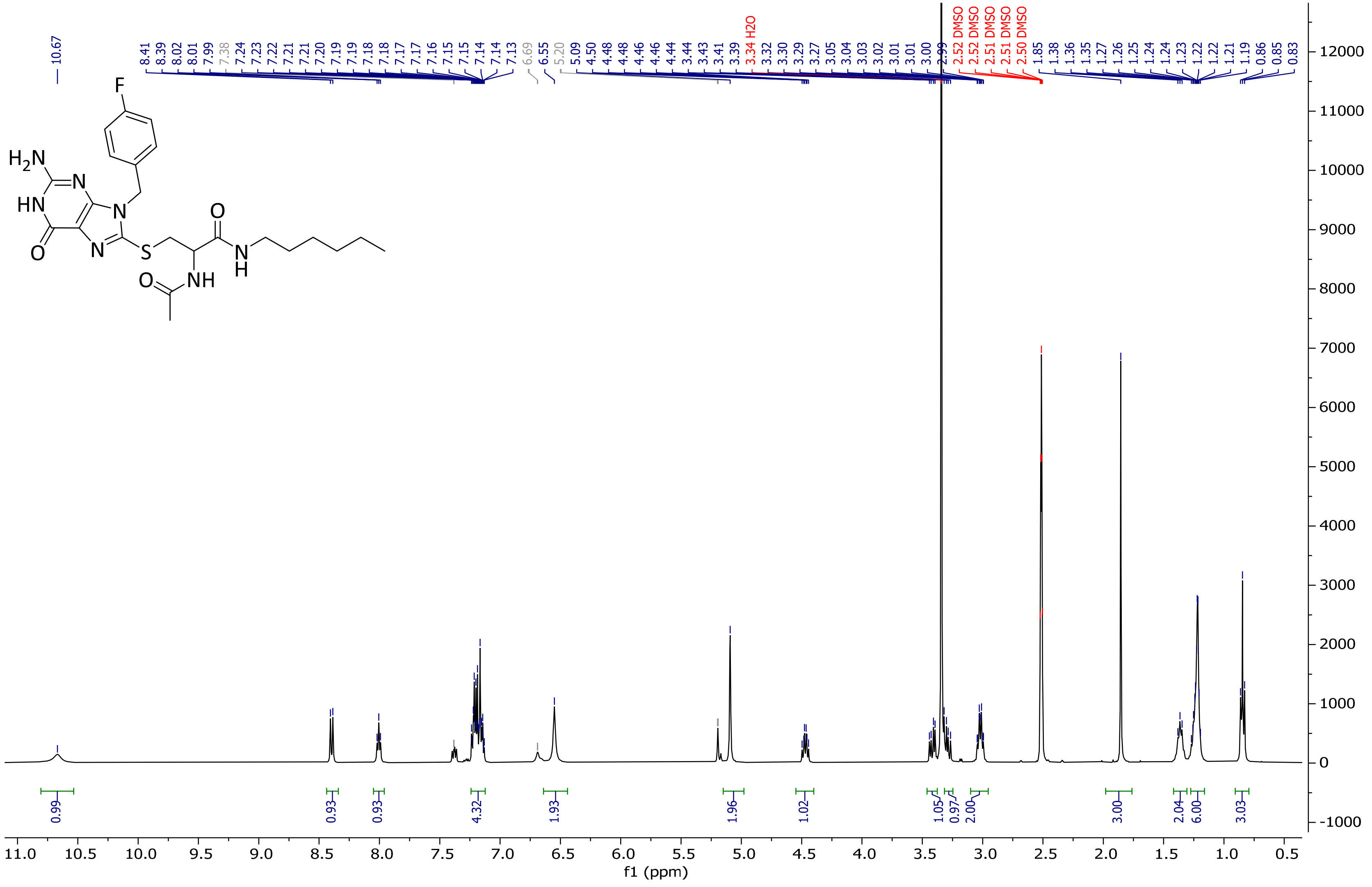

Figure S13. ^{1}H NMR (400 MHz) spectrum of 2-acetamido-3-((2-amino-9-(4-fluorobenzyl)-6-oxo-6,9-dihydro-1H-purin-8-yl)thio)-*N*-hexylpropanamide (7) in DMSO-d_6

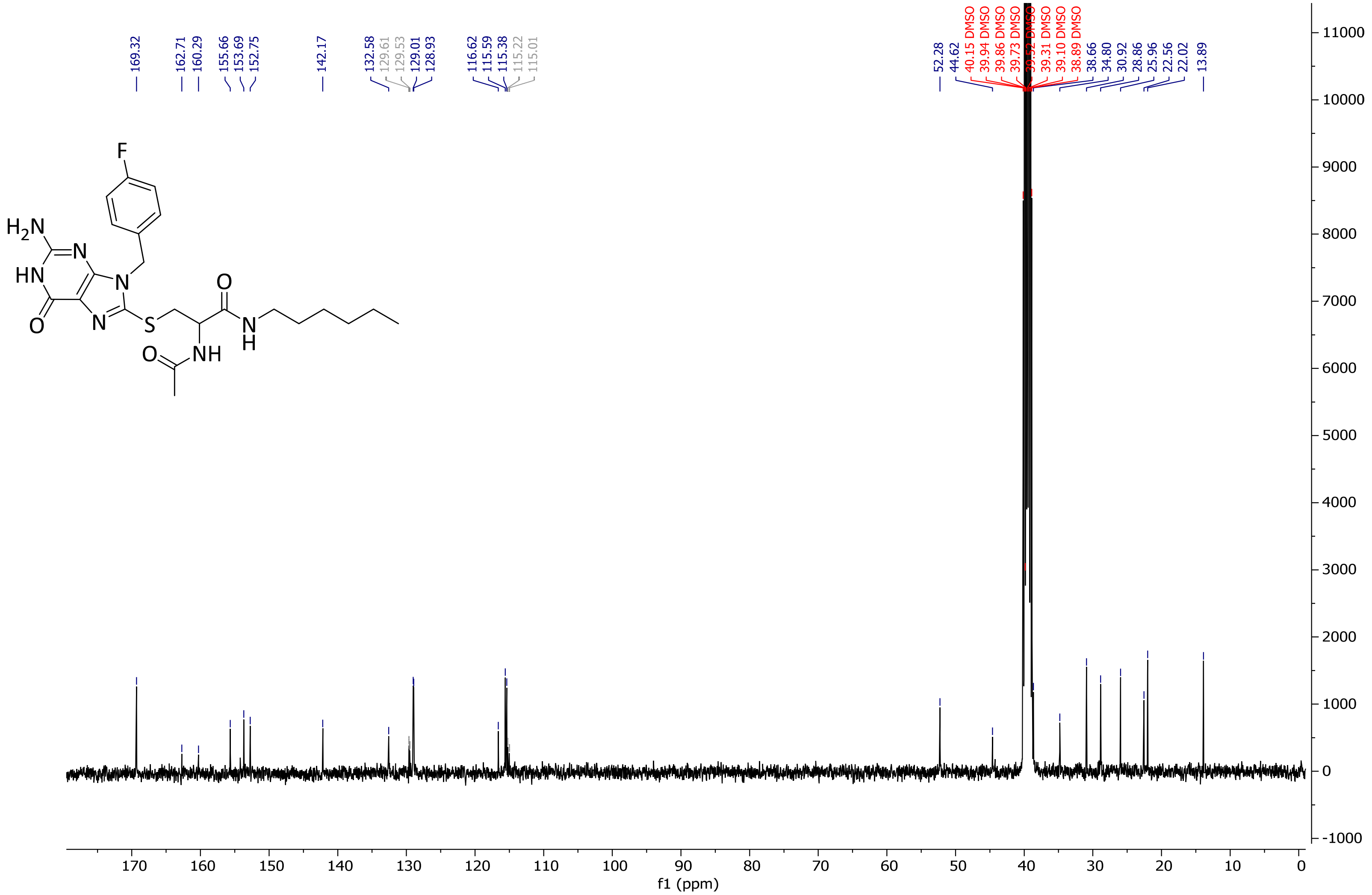

Figure S14. ^{13}C NMR (101 MHz) spectrum of 2-acetamido-3-((2-amino-9-(4-fluorobenzyl)-6-oxo-6,9-dihydro-1H-purin-8-yl)thio)-*N*-hexylpropanamide (7) in DMSO-d_6

Confocal microscopy images of KP4 cells

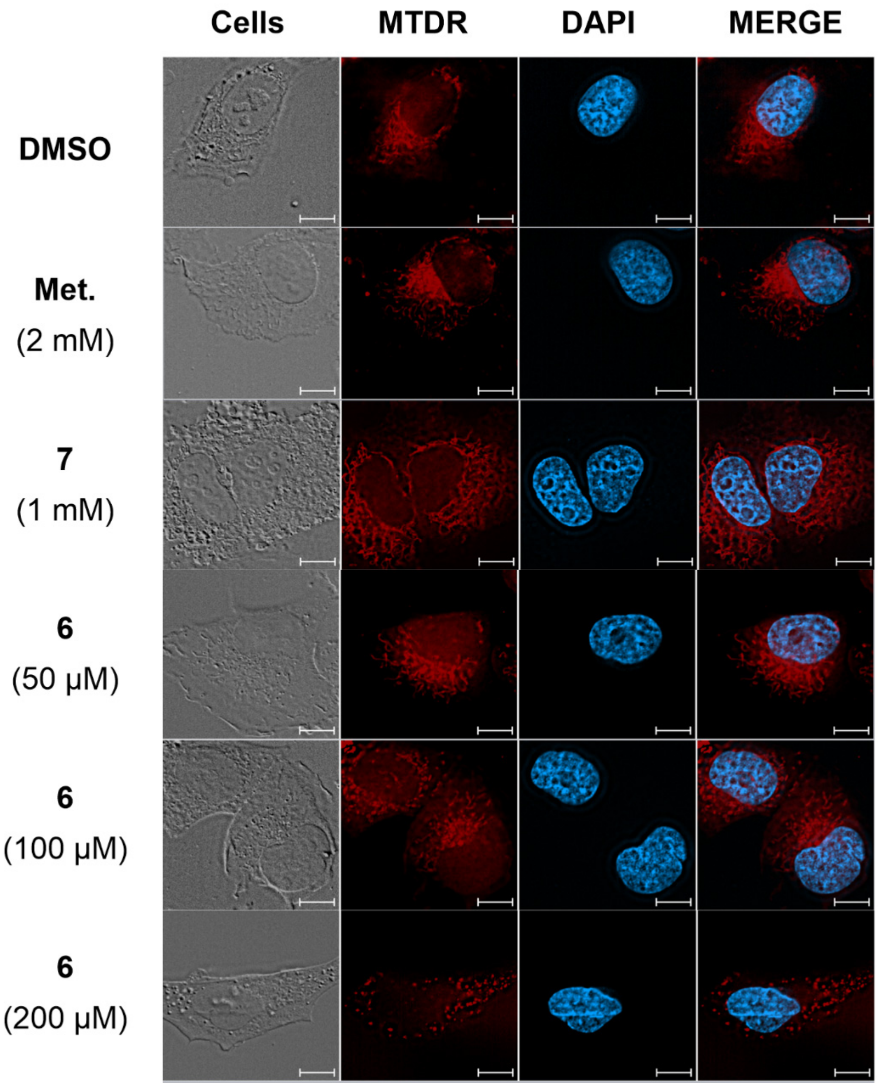

Figure S15. Confocal microscopy images of K4 pancreatic cancer cells following treatment With AUTAC-Biguanide **6** and its Controls; Staining of mitochondria with the Mitotracker® Deep Red FM (MTDR) fluorescent probe and the nuclei with the fluorescent dye DAPI.
